# Dual Porosity Protein-based Scaffolds with Enhanced Cell Infiltration and Proliferation

**DOI:** 10.1038/s41598-018-33245-w

**Published:** 2018-10-05

**Authors:** Morteza Rasoulianboroujeni, Nasim Kiaie, Fahimeh Sadat Tabatabaei, Amir Yadegari, Farahnaz Fahimipour, Kimia Khoshroo, Lobat Tayebi

**Affiliations:** 1 Marquette University School of Dentistry, Milwaukee, WI USA; 2 Division of Pharmaceutical Sciences, School of Pharmacy, University of Wisconsin-Madison, Madison, WI USA; 3 Department of Tissue Engineering, Amirkabir University of Technology, Tehran, Iran; 4 Department of Dental Biomaterials, School of Dentistry, Shahid Beheshti University of Medical Sciences, Tehran, Iran

## Abstract

3D dual porosity protein-based scaffolds have been developed using the combination of foaming and freeze-drying. The suggested approach leads to the production of large, highly porous scaffolds with negligible shrinkage and deformation compared to the conventional freeze-drying method. Scanning electron microscopy, standard histological processing and mercury intrusion porosimetry confirmed the formation of a dual network in the form of big primary pores (243 ± 14 µm) embracing smaller secondary pores (42 ± 3 µm) opened onto their surface, resembling a vascular network. High interconnectivity of the pores, confirmed by micro-CT, is shown to improve diffusion kinetics and support a relatively uniform distribution of isolated human dental pulp stem cells within the scaffold compared to conventional scaffolds. Dual network scaffolds indicate more than three times as high cell proliferation capability as conventional scaffolds in 14 days.

## Introduction

Engineered constructs, known as 3D scaffolds, resembling the natural Extra Cellular Matrix (ECM) and supporting cell functions are being widely investigated for tissue regeneration purposes. An ideal scaffold should facilitate nutrient supply and waste removal while enabling cell adhesion, growth, proliferation and migration. Although scaffold ingredients and composition play an important role in this matter, tailoring the inner architecture of the scaffold toward imitating the function of natural tissues can further improve cellular function and activity^1,2^.

Controlling the pore size distribution and covering different length scales can considerably influence the scaffold performance. While very small pores allow for molecular transport essential for nutrition, waste removal and signaling, bigger pores facilitate cell migration and capillary formation, as well as the incorporation of nerves and blood vessels^3,4^. This is particularly important when critically sized defects are taken into consideration when nutrient supply is a problem and attempts are being made to improve vascularization^5^.

In the journey toward developing an efficient scaffold, dual porosity scaffolds with a primary interconnected network of big pores and a secondary interconnected network of smaller pores are becoming the matter of interest. Some studies have combined two different methods of fabrication to produce multiple pore sizes or geometries. Examples include the combination of 3D-printing/salt leaching^6^ and micro-molding/porogen templating^7^. Multi-material scaffolds consisting of a microporous structure made of a polyester, for example, and a micro or nanoporous structure obtained by filling the primary structure with a hydrogel have also been suggested as dual porosity scaffolds^8^. Dipping a metallic foam into a polymer solution followed by cross-linking of the coated polymer and etching the metallic template away is another suggested method to produce dual network scaffolds resembling vascular networks observed in tissue^9^. Lastly, a surface gelling mechanism has been exploited to create small pores within the walls of bigger pores of a polyurethane-based scaffold made by inkjet printing^10^. In addition to the high cost of equipment and complexity of procedure, and despite potentially yielding the desired structure, the mentioned methods are not applicable to sensitive biomolecules like proteins.

Amongst various fabrication methods, freeze-drying is of great importance as it allows fabrication of protein-based scaffolds where damaging process parameters and elements such as heat or solvents need to be avoided. Even though freeze-drying has been used in combination with other methods to produce dual porosity polyester-based scaffolds^11^, production of dual porosity protein-based scaffolds using this method has not been well concentrated. This method includes freezing the structure followed by sublimation of the ice crystals and desorption of non-frozen bound water under vacuum. The porosity could be controlled by managing the freezing process. Despite the advantages, this expensive method suffers from some weaknesses. The long drying time due to limited diffusion restricts the size of the scaffolds that can be fabricated through this method^12–14^. Furthermore, insufficient control over the pore size, despite the attempts made by many authors to control the ice crystal growth on one side and scaffold shrinkage and structural deformation on the other side, remain unaddressed^15–18^. Hence, developing a method that offers freeze-drying advantages but eliminates its drawbacks is highly desired. Such a method is not only expected to facilitate production of large scaffolds with finely tuned pore size, but also to grant the co-existence of small and big pores in the form of dual interconnected networks.

In this study, we suggest a new facile method for fabrication of large gelatin-based scaffolds with such an architecture. We combined foaming and freeze-drying methods to not only abolish the shortcomings of the conventional freeze-drying protocol, but to also achieve a highly porous scaffold with dual interconnected networks, which is reported here for the first time. The obtained structure supports a relatively uniform distribution of the cells throughout the scaffold and triples the proliferation capacity.

## Results

### Morphology of the primary pores

**Figure 1:**
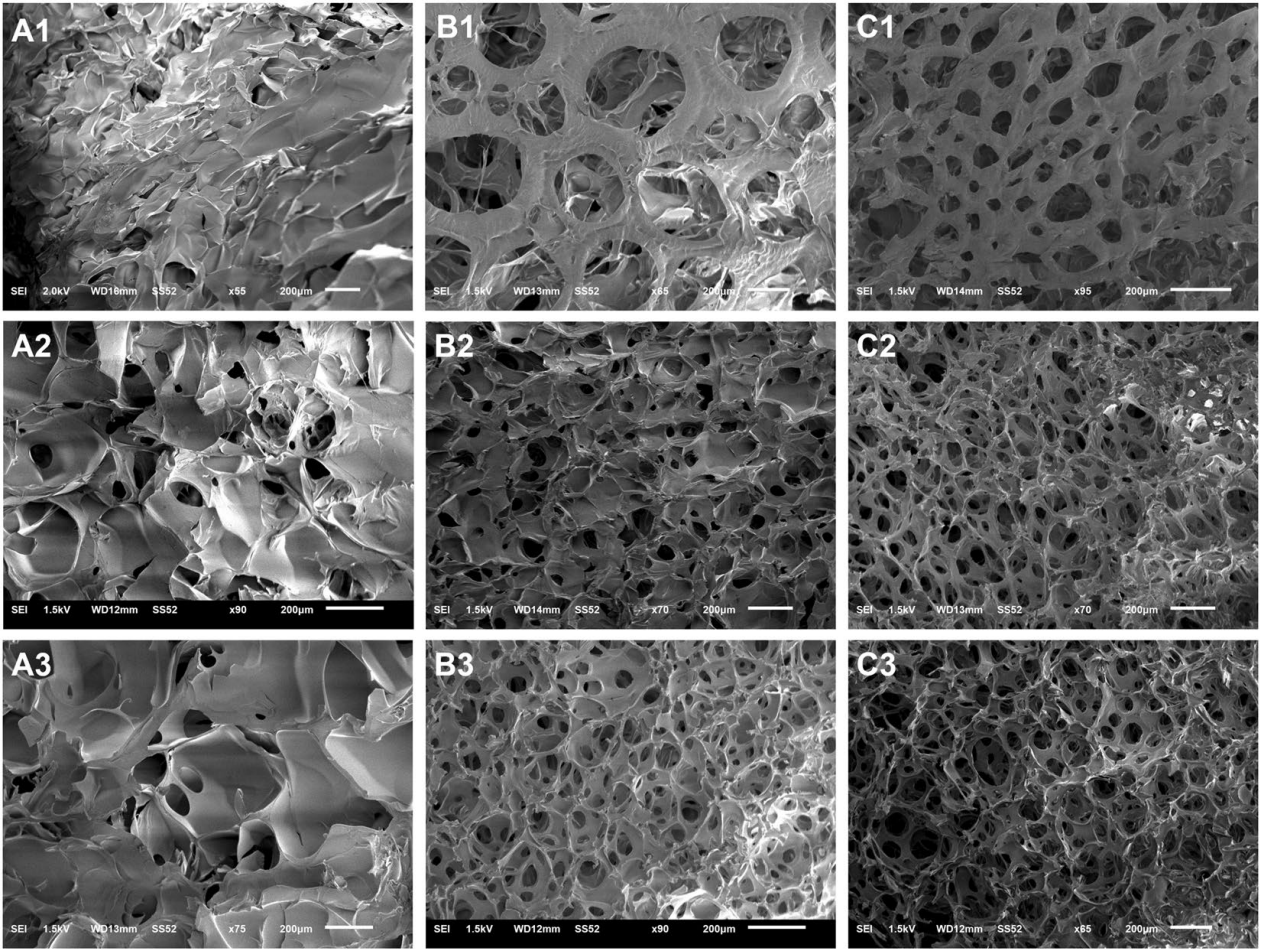
Scanning electron microscope images of scaffolds made of Gelatin 5% w/v foamed at 0 (**A1**), 500 (**B1**) or 1500 (**C1**) rpm, 10% w/v foamed at 0 (**A2**), 500 (**B2**) or 1500 (**C2**) rpm, 15% w/v foamed at 0 (**A3**), 500 (**B3**) or 1500 (**C3**) rpm.

The morphology of primary pores was investigated using scanning electron microscopy (SEM). Figure 1 shows the SEM images of the primary porous architecture of the scaffolds made using various concentrations of gelatin solution (5–15% w/v, rows 1–3 in Fig. 1) and agitation speeds (0–1500 rpm, columns A-C in Fig. 1). The agitation and molding was performed at 40 °C, well above the gelation temperature of all formulations (Supporting Information, Figs S1 and S2). Scaffolds prepared via conventional freeze drying, i.e. without foaming (0 rpm, Fig. 1-As), despite yielding porous structures are not comparable to the foamed ones (Fig. 1-B and Cs) in terms of order, uniformity and interconnectivity of the primary pores. The foamed samples present more homogenous primary structures, better mimicking the architecture of the natural tissues like bone. The obtained structure was positively influenced by increasing both the gelatin concentration and agitation speed. When 5% w/v gelatin was used, agitation at neither low (500 rpm) nor high (1500 rpm) speeds could produce ideal morphology as seen in Fig. 1-B1 and C1, respectively. In contrast, high concentration of gelatin, i.e. 15% w/v (Fig. 1-B3 and C3), gives high-quality primary structure even at low speeds. Interestingly, moderate concentration, i.e. 10% w/v, can produce acceptable morphology only when high speed is employed (Fig. 1-C2). Agitation speeds as low as 500 rpm are unable to induce adequate bubbling in 10% w/v gelatin and the obtained morphology is similar to the conventional samples (Fig. 1-B2). These phenomena suggest the simultaneous significance of concentration and speed.

### Porosity, mechanical properties, shrinkage and deformation

**Figure 2:**
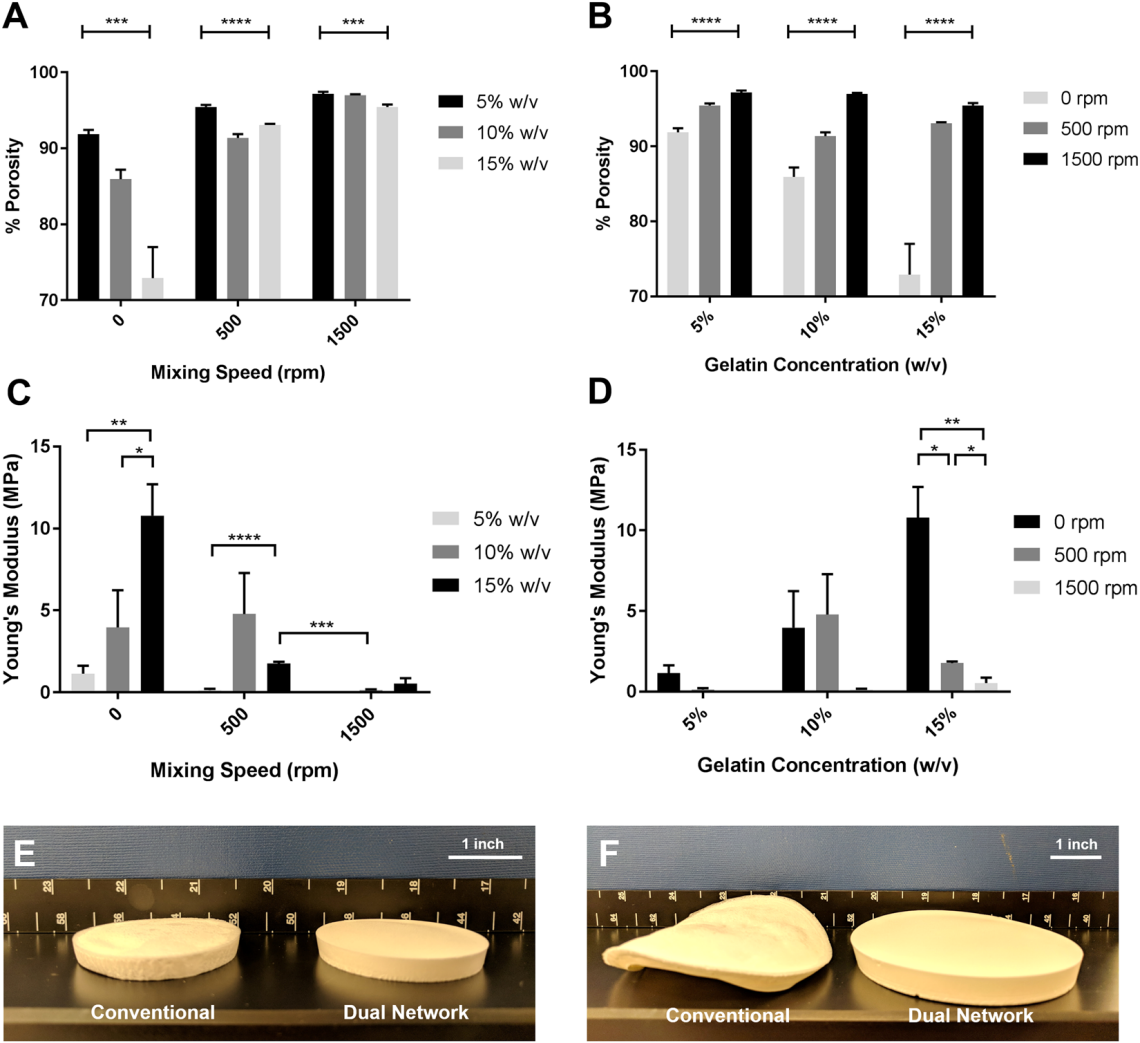
Effect of agitation speed and gelatin concentration on the porosity (**A**,**B**) and modulus (**C**,**D**) of the conventional and foam scaffolds. The outcome of freeze-drying in terms of deformation and shrinkage can be seen for medium size (**E**) and large size samples (**F**).

The effect of gelatin concentration and agitation speed on the porosity and mechanical properties of the final scaffolds were also examined. The porosity (pore volume) and Young’s modulus were also found to be influenced by both concentration and speed of agitation as shown in Fig. 2A–D. As seen in Fig. 2A, for each agitation speed, porosity decreases with increasing concentration, with the exception of 10% w/v gelatin at 500 rpm due to its intermediate structure (see Fig. 1-B2). According to Fig. 2B, porosity also increases with increasing speed for all concentrations (p < 0.0001) and, in general, the effect of agitation speed is more significant than concentration. In regards to mechanical properties, compressive modulus increases with the increase of concentration in conventional scaffolds (p < 0.05), as expected (Fig. 2C). According to Fig. 2C, at 500 rpm, the gelatin concentration had a significant effect on the modulus of the scaffolds (p < 0.0001). Among all the samples prepared at this agitation speed, sample 10%-500rpm had the highest modulus again maybe because of its intermediate structure (see Fig. 1-B2). When agitated at 1500 rpm (Fig. 2C), the sample 5%-1500rpm was too weak to endure mechanical testing, and for the other two concentrations, no significant difference in modulus was observed (p > 0.05). Considering the effect of agitation speed at a certain concentration (Fig. 2D), only at the high concentration was the effect of agitation speed found to be significant (p < 0.05). In this case, the modulus decreased with the increase of agitation speed.

Among all the samples, 15%-500rpm (high concentration, low speed) was found to have the desired primary structure (local interconnected pores observed in SEM images), high porosity (>90%) and significantly superior modulus (>1 MPa) compared to 10%-1500rpm (p < 0.001) and 15%-1500rpm (p < 0.05) and was thus selected for additional analysis. This sample has the minimal deformation compared to the conventional scaffolds and presents a shrinkage of less than 5%, as illustrated in Fig. 2E,F. However, as expected, this sample is weaker than the corresponding conventional one i.e. 15%-0rpm simply because of significantly higher porosity (93% vs. 73%). Interestingly, the compressive modulus of the optimal dual network scaffold is not significantly different (p > 0.05) from that of conventional scaffolds prepared using lower concentrations i.e. 5%-0rpm and 10%-0rpm although the latter’s porosity is significantly lower (p < 0.01). It can be presumed then that the stiffness of dual network scaffolds can be rectified by using higher concentrations of gelatin whereby substantial reduction of porosity can be avoided.

### Micro-architecture and diffusion kinetics

**Figure 3:**
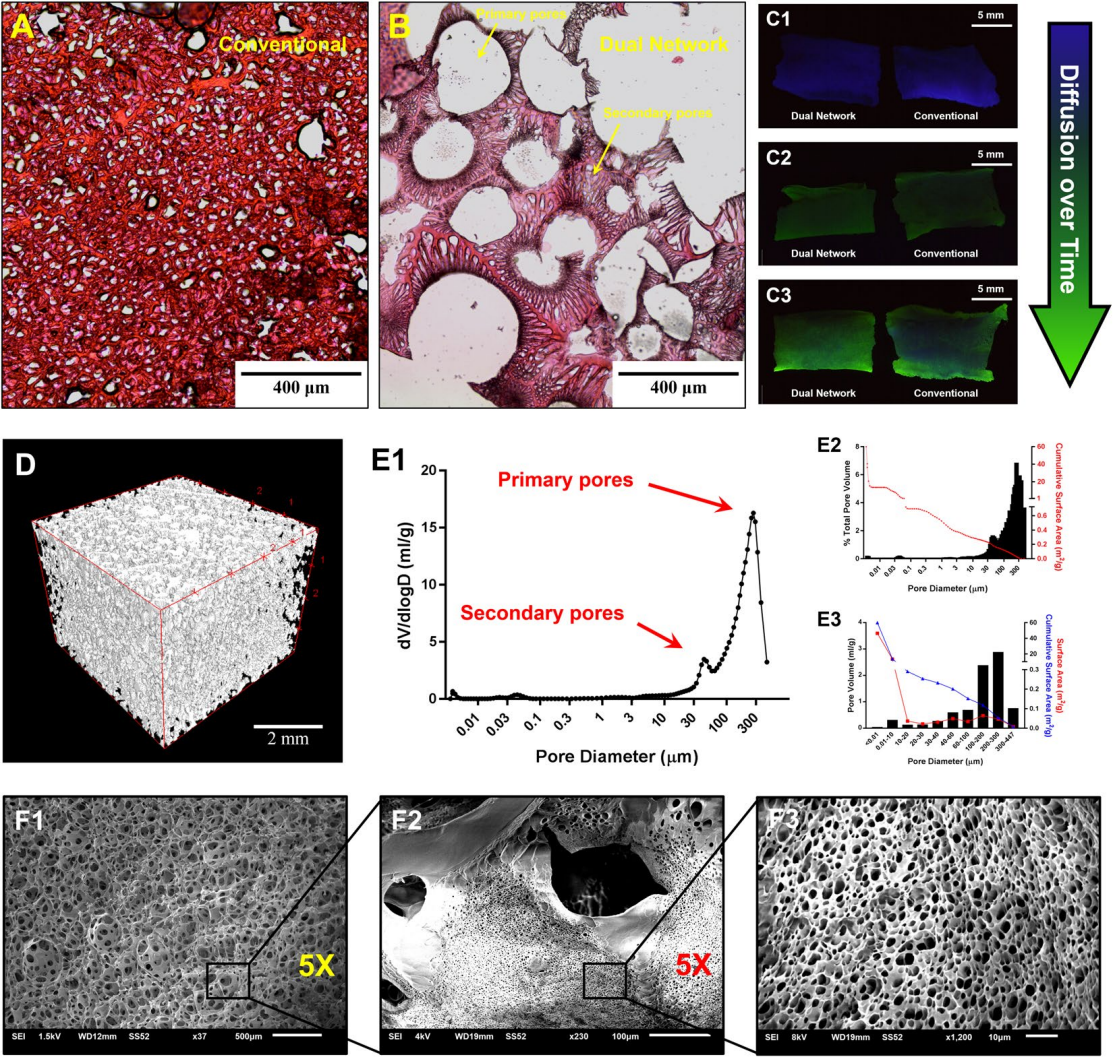
The light microscope images of the cross-sections of conventional (**A**) and dual network (**B**) scaffolds prepared and stained using standard histology/tissue processing protocols. The diffusion of fluorescein sodium salt as the model substance into the conventional and dual network scaffolds after 0 (**C1**), 0.5 (**C2**) and 1.5 (**C3**) h immersion in 10 µg/ml aqueous solution. Micro-CT 3D image of the dual network scaffold (**D**). Pore size distribution profile of the dual network scaffold (**E1**), % total pore volume and cumulative surface area as a function of pore size (**E2**) as well as pore volume, surface area and cumulative surface area for different ranges of pore size (**E3**) obtained by mercury intrusion porosimetry. The SEM images of primary pores (**F1**), porous wall of a primary pore (**F2**) and the opening of the secondary pores (**F3**) of the dual network scaffold.

To observe the differences between the micro-architectures of the dual porosity scaffolds and conventional scaffold (15%-0rpm), standard histological processing was employed. Figure 3A,B illustrate the cross-sections of the conventional and dual network scaffold, respectively. While the conventional scaffold presents a random network of pores governed by the formation and growth of ice crystals, the newly developed one offers a fully interconnected dual network in the form of big primary pores embracing smaller secondary pores opened onto their surface, resembling vascular networks. The appearance of the secondary structure reminds of ice crystal growth on the surface of a bubble. The large pores form as a result of the bubbling process, while the smaller pores arise due to the development and sublimation of ice crystals. Such an architecture allows for better mass transport management (i.e., nutrients, gases, and waste) as shown in Fig. 3C wherein the diffusion of the fluorescent dye, fluorescein sodium salt, as a function of time has been illustrated. The hydrophilic dye as the model substance may better mimic the diffusion kinetics of nutrients and waste rather than gases. To investigate the accessibility of central regions of the scaffolds when the nutrient supply/waste removal is being managed in the surrounding environment, the scaffolds were immersed into the dye solution, kept for a certain time, cut into half and imaged on cross-section. As seen in Fig. 3-C1, both scaffolds display autofluorescence (blue color) at the beginning of the experiment before being exposed to the dye solution. Over time, the blue areas are replaced with green (Fig. 2-C2) which shows the presence of fluorescent dye. As seen in Fig. 2-C3, the conventional scaffold presents more blue and less green areas compared to the dual network one, suggesting facilitated diffusion by the latter. Micro-CT 3D imaging also confirms the interconnectivity of the pores in dual network scaffolds and further supports the facilitated diffusion (Fig. 3D). The Euler characteristic and connectivity density (Conn. D) of the developed scaffold are −2974 and 24.8 mm^−3^, respectively. Further characterization of dual network scaffold using mercury intrusion porosimetry exhibits quite interesting data (Fig. 3E). Bimodal distribution with maximums at 243 ± 14 and 42 ± 3 µm (Fig. 3E1) supports the dual network formation and is in strong agreement with cross-sectional images. While the size of majority of the primary structure pores fall within the range of 100–300 µm, the secondary structure pore size distribution is very broad (Fig. 3-E2 and E3). At some spots, the opening, not necessarily the diameter, of the secondary pores on the wall of the primary pores is smaller than 5 µm as seen in Fig. 3F. The total surface area of the dual network scaffold is 59.9 m^2^/g. Figure 3-F1 displays the primary architecture of the optimum scaffold. Increasing the magnification, the openings of secondary pores on the walls of primary pores can be seen (Fig. 3-F2). Further magnification allows obvious observation of openings of the secondary porosity (Fig. 3-F3)

### Attachment, distribution and proliferation of isolated dental pulp stem cells on the scaffolds

**Figure 4:**
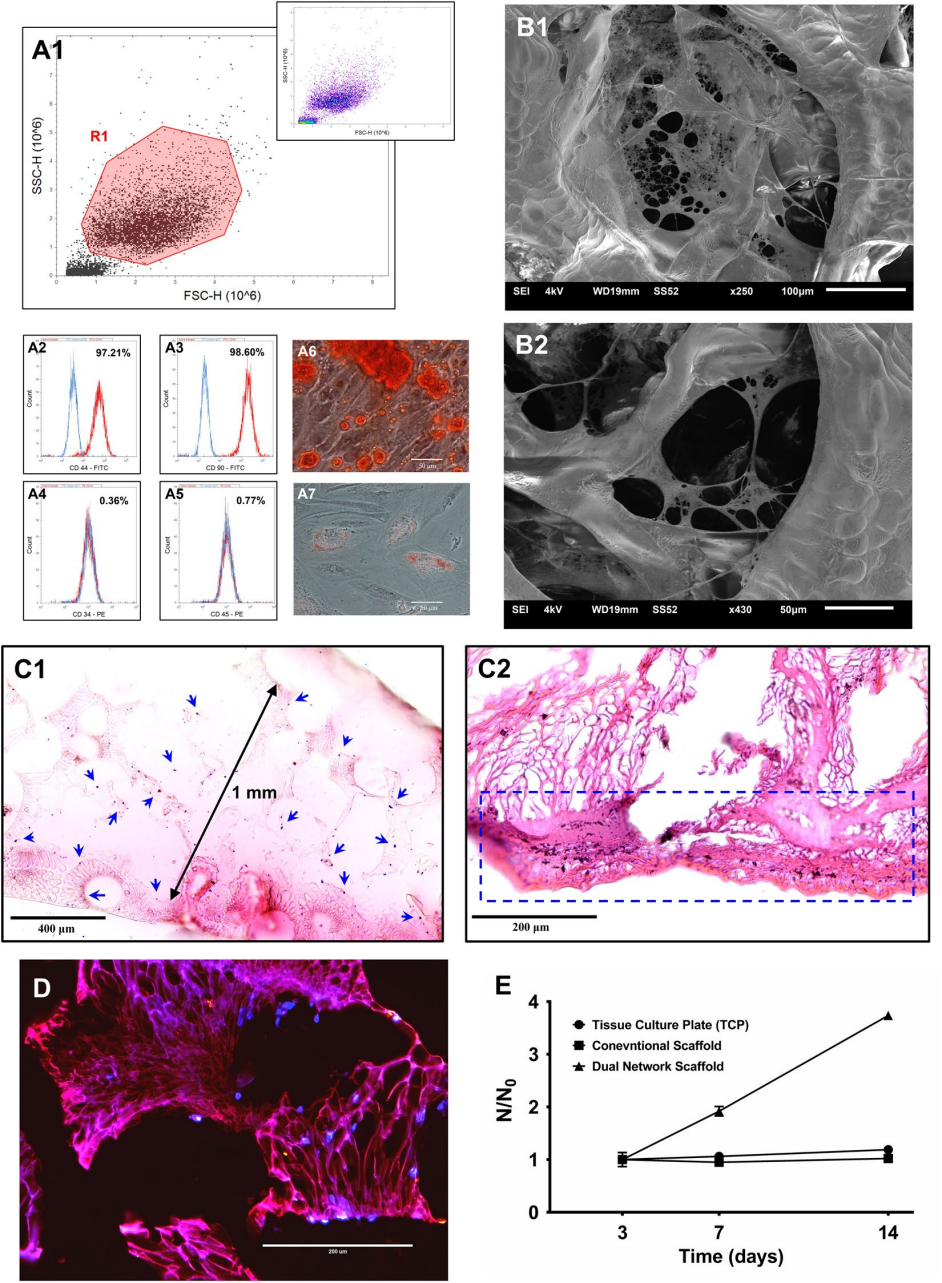
Isolation of DPSCs was confirmed by flow cytometric analysis (**A**); for the selected population (**A1**), the expression of CD44 (**A2**), CD90 (**A3**), CD34 (**A4**) and CD45 (**A5**) was investigated. The isolated cells exhibited alternatively osteogenic (**A6**) or adipogenic (**A7**) differentiation. SEM images revealed DPSCs adhesion to the dual network scaffold (**B**) at low (**B1**) and high (**B2**) magnification. H&E staining corroborated uniform distribution of the cells (blue arrows) all over the dual network scaffold (**C1**) while revealing superficial growth (dashed box) on the conventional ones (**C2**). Immunohistochemical staining of the dual network scaffolds (**D**). The cell proliferation rate reported as the cell number normalized to the initial number of cells (N/N_0_) on dual network and conventional scaffolds as well as tissue culture plate (**E**).

Isolated dental pulp stem cells (DPSCs) were used to investigate cell attachment, proliferation and distribution in the scaffolds. Adequate isolation of DPSCs from the human third molar teeth can be confirmed by flow cytometry and differentiation capacity of the isolated cells (Fig. 4A). DPSCs are assumed to be positive for the CD44 and CD90 markers while being negative for CD34 and CD45 (hematopoietic markers). As expected, more than 97% of the cells display the presence of CD44 and CD90 and absence of CD34 and CD45 antigens (Fig. 4A1–5). The presence or absence of certain antigens on the surface of selected cell population (Fig. 4-A1) can be quantified using the extent of peak shift in the flow cytometer histograms (Fig. 4A2–A5). On the other hand, DPSCs are capable of osteogenic or adipogenic differentiation if subjected to appropriate media. Osteogenic and adipogenic differentiation capacity of the isolated cells can be confirmed by Alizarin Red S (Fig. 4A6) and Oil Red O (Fig. 4A7), respectively. The flow cytometry results along with differentiation capacity support efficient isolation of DPSCs. The isolated DPSCs were then used to seed the scaffolds and evaluate their biological performance. The seeded DPSCs grow within the pores of the scaffold with their multiple cell processes branching from one side to another, forming an adherent plexus all over the pore as illustrated in Fig. 4B. It can be vividly seen that the DPSCs have attempted to anchor their cytoskeletal projections (lamellipodia and Filopodia) onto the pore surface (Fig. 4-B1 and B2). The Hematoxylin & Eosin (H&E) (Fig. 4C1) and immunohistochemical (Fig. 4D) staining of the foamed sample both not only confirm the existence of dual network again, but also reveal the uniform distribution of the cells all over the scaffold. The cells have covered a depth of at least 1 mm within the scaffold (Fig. 4C1) and seem to line along the walls of primary pores in the scaffold where the openings of the secondary pores are located. In contrast, cells have grown mostly on the surface of the conventional scaffolds (Fig. 4C2). The cell proliferation results (Fig. 4D) suggest more than three times improvement in proliferation capability of the cells seeded on dual network scaffolds compared to both conventional scaffolds and tissue culture plate that can be because of 3D growth of the cells on the former construct.

## Discussion

In the suggested method, the gelatin aqueous solution is agitated at a specific speed to produce a foamy liquid that might be very stable depending on the concentration and speed. The foamy liquid is molded, freeze-dried, cross-linked and then freeze-dried again to obtain the final scaffolds. This method is applicable to many proteins because of their emulsifying properties and foaming ability owed to their complex structure. The ability to stabilize an emulsion or trap gaseous bubbles is originated from the amphiphilic nature of proteins, i.e. co-existence of hydrophilic and hydrophobic domains in the structure. It is known that proteins have a lower surface tension compared to water^19^ and can thus reduce the surface tension of the solution^20^. In addition to surface hydrophobicity, the foaming ability of a protein can also be influenced by diffusion coefficient and isoelectric point. The diffusion coefficient affects the ease of migration of a protein molecule to the liquid-air interface, while being near the isoelectric point facilitates the protein adsorption on the interface through minimizing the electrostatic interactions^21^. Therefore, Type A gelatin is a better emulsifier and foaming agent compared to Type B because of its closer-to-neutral isoelectric point (7–9 vs 5)^22^. Adsorption of gelatin molecules onto the gas bubbles is a thermodynamically favorable process because of immediate dehydration of the hydrophobic interfaces^21^. It has been shown that covalent attachment of hydrophobic groups to gelatin makes its foaming ability comparable to that of sodium dodecyl sulfate (SDS)^23,24^.

The suggested method produces a dual network of big primary pores embracing smaller secondary ones. High concentrations of gelatin and high speeds of agitation were found to yield more ordered and interconnective primary pores. The morphology of the scaffold is indeed governed by the quality and stability of the precursor, which in turn is influenced by the kinetics of foaming/aging process (Supporting Information, Figs S3–S5). Higher concentrations and speeds are in favor of foam stability due to increased stiffness and viscoelasticity of the lamella making the bubbles resilient to rupture^24^, and improved transportation of protein molecules onto lamella facilitating interaction with bubbles^25,26^, respectively. In addition, increasing concentration means lowering of surface tension^27^ and ameliorating the shear rate distribution^28^. On the other hand, diffusion of gas molecules due to Laplace pressure between bubbles, known as coarsening or Ostwald Ripening, and drainage of proteins from lamellae because of gravitational forces, recognized as two important aging mechanisms leading to complete collapse of the foam over time^29^, are also affected by concentration and speed.

To fill the large defects, the biggest claimed size that can be fabricated through freeze-drying with good control over the architecture has been 1–1.5 cm in diameter and a few millimeters in height^30–33^. The percentage of shrinkage has been reported to be 35%^34^. Such drawbacks arise from the limited heat and mass transfer in the conventional freeze-drying method. The suggested method, in turn, allows facilitated diffusion of water molecules and sublimation of ice crystals without damaging the structural integrity of the scaffold. Indeed, the trapped bubbles not only reduce the diffusion resistance compared to condensed phase, but also provide a high surface area for mass transfer, ultimately improving the drying rate and quality^35^. Using this strategy, the deformation could be avoided and the shrinkage reduced to less than 5%, as pointed out in the “Results” section.

The suggested method yields a dual network scaffold wherein the large pores form as a result of the bubbling process, and the smaller pores arise due to the development and sublimation of ice crystals. One should note that the pore size of secondary network can be controlled by controlling the size of ice crystals which in turn is governed by the freezing rate. Using the mentioned process parameters, a primary pore size of 243 ± 14 µm and a secondary pore size of 42 ± 3 µm were obtained that are in the range of values used by many studies^6,8,11,36^. Such an architecture allows for better mass transport management (i.e., nutrients, gases, and waste) due to enhanced interconnectivity. While both large primary pores and small secondary pores can facilitate mass transport, the presence of the small pores can greatly enhance pore interconnectivity as they form channels between the large pores^11^. The highly negative value of Euler characteristic confirms the observation about improved diffusion of fluorescent dye. Euler characteristic, a measure of 3D connectivity, becomes more negative as more connections exist between pores and is close to zero in the case of poor connectivity^37^. It should be noted that for a more accurate evaluation of interconnectivity, further consideration regarding the weaknesses of this parameter is needed. The number of components and the number of enclosed cavities as well as the edge effects can mislead the estimation of connectivity and need to be controlled^38^. The big value of Conn. D, the number of loops per unit volume of material, compared to trabecular bone (5.68 mm^−3^) and the maximum reported value for bone scaffolds, i.e. 6 mm^−3^ ^39,40^, is also of great importance. The total surface area of the dual network (59.9 m^2^/g) is comparable to catalyst supports with intermediate surface area, zeolite^41^ and carbon nanotubes^42^. Even though reducing the pore size can increase the available surface area, the ability of larger pores to facilitate cell infiltration can override the beneficial effect of greater initial cell attachment surface areas provided by smaller pores^43^. The suggested dual porosity scaffold wherein small pores are present in the walls of larger ones allows for better cell attachment due to higher available surface area while retaining the high cell infiltration through large pores. Such a high available surface area and enabled cell infiltration along with efficient nutrient delivery due to facilitated diffusion makes the dual network scaffold an appropriate substrate for cell attachment, growth, proliferation and immigration. The optimum reported pore size range varies with the type of cell or tissue^44–46^. There are many potential applications for the primary pore size range obtained in this study. For instance, using multilayered agent-based model simulation, it has been shown that large pore sizes of approximately 160 to 270  μm facilitate angiogenesis throughout scaffold^47^. Optimal cell proliferation and infiltration occurred in Collagen-GAG scaffolds when the mean pore size around 300  μm^43^. Lastly, different studies have suggested the importance of having pore sizes around 300  μm for osteogenesis to occur^43,48^. Employing the optimal pore size range, a balance between the advantages and disadvantages of varying the scaffold’s pore size can be established. Pore size or porosity of the scaffold can influence nutrient supply, gas diffusion, metabolic waste removal, cell attachment and intracellular signaling^45,46,49^. Therefore, co-existence of different size scale pores may improve cellular activity. While very small pores facilitate molecule transport essential for nutrition and signaling, improve interconnectivity and increase available surface area for cell attachment, larger ones allow for cell infiltration, migration and capillary formation^3^. Co-incorporation of various porosities may result in a more uniform distribution of the cells and superior cell proliferation as obtained through dual porosity scaffolds in this study.

## Conclusion

The technique developed in this study allows the fabrication of large, highly porous protein-based scaffolds with minimum shrinkage. Coexistence of primary big pores and secondary small pores in the form of individual interconnected networks can support cell attachment/growth and nutrient/gas transport. More uniform distribution of isolated human dental pulp stem cells along with more than three times as high cell proliferation capability in two weeks compared to conventional scaffolds, indicates the superiority of the dual network scaffolds.

## Experimental Procedure

### Materials

Gelatin type A from porcine skin was purchased from Sigma (USA). 1-Ethyl-3-(3-dimethylaminopropyl) carbodiimide (EDC) and N-Hydroxysuccinimide (NHS) were obtained from TCI America (USA) and Alfa Aesar (USA), respectively.

### Scaffold preparation and shrinkage monitoring

Different concentrations of gelatin aqueous solution (5%, 10%, 15% (w/v)) were prepared by dissolving gelatin in DI water at 50 °C. Each solution was agitated at 500 or 1500 rpm using a mechanical mixer (IKA, USA) for 15 min while maintaining the temperature at 40 °C. The obtained foam was molded in petri-dishes (D = 50 mm, H = 7 mm) for each group, transferred to −80 °C and kept overnight. The same protocol was applied to the gelatin solutions of the same concentration with the first agitation step being skipped to follow conventional protocols. Samples were then freeze-dried at −52 °C, 1 Pa for 36 h followed by 12 h secondary drying at 25 °C, 1 Pa. The obtained foams were cross-linked using 4 mg/ml EDC, 0.5 mg/ml NHS in ethanol 90% (v/v) at 4 °C for 48 h. 20 ml of cross-linking solution was used per scaffold. After cross-linking, samples were washed for 4 hours using DI water to remove residual cross-linker. Samples were freeze-dried again after washing according to the aforementioned protocol.

The shrinkage after freeze-drying was evaluated through measuring the dimensions of the scaffolds using a digital caliper and comparing them to those of the mold. To monitor the shrinkage and deformation, molds of three different sizes were used: D × H = 33 × 11, 50 × 7 and 85 × 12 mm.

### Morphology of the scaffolds’ pores

Scanning electron microscopy (SEM, JEOL-JSM6510, Japan) was used to observe the pore structure of each scaffold. Samples were sputter-coated with a layer of gold prior to imaging. Imaging was performed using secondary electron mode at different accelerating voltages (1.5 to 8 kV) and magnifications.

### Porosity and Mechanical properties

Porosity or pore volume of the samples was measured using a solvent displacement method. Ethanol was selected as the solvent as it infiltrates into the scaffolds’ pores without swelling or shrinking the matrix. The porosity of the scaffold was calculated using the following equation:

1 $$ \% \,porosity=\frac{{W}_{2}-{W}_{1}}{{W}_{2}-{W}_{3}}\times 100\,$$

where W_1_ is the weight of the sample in air, W_2_ is the weight of the sample with liquid in pores, and W_3_ the weight of the sample suspended in ethanol.

To measure the compressive young’s modulus for each scaffold, a universal testing machine (UTM, AGS-X, Shimadzu) equipped with a 1 kN load cell was employed. Cubes of approximately 5 × 5 × 55 mm3 were cut from each sample and used. The experiments were conducted at the constant compression speed of 1 mm/min until failure. The measurements were performed in triplicate, averaged and reported.

The scaffold with optimal morphology and mechanical properties was selected based on the data obtained through SEM, porosity and modulus measurement.

### Pore size distribution and 3D reconstruction of the optimal scaffold

The pore size distribution, pore volume distribution, total pore volume and surface area of the selected scaffold as well as the total surface area were quantified using a mercury intrusion porosimeter (Porous Materials Inc., USA). The pore size distribution can be obtained by measuring the sensitivity of the intruded volume to pressure change (dV/dlogP). For surface area measurement, the pores are assumed to be cylindrical.

3D sketch of the scaffold was constructed by ImageJ software (version 1.5i, National Institute of Health, USA) through stacking of virtual cross section slices obtained by micro computed tomography (micro-CT). The micro-CT system consisted of a Hamamatsu L9181–02 micro-focal X-ray source and a flat panel X-ray detector (Varian 2520DX). A micro-positioning stage between the source and detector allows specimens to be manipulated in space with four degrees of freedom. Micro-CT reconstruction was implemented using the Varian Cone-Bean CT reconstruction software. The CT scan consisted of 400 step and shoot projections with a square trajectory. The tube current time product was 19.2 mAs. The images were acquired at 100 kV and 80 μA, with 0.5 second exposure per view angle. The images were reconstructed on to 20-micron cubed voxels. The 3D image of the scaffold was produced using 3D Viewer plugin after adjusting the threshold by Iso Data method. The Euler characteristic and connectivity density (number of trabeculae per unit volume) were calculated by means of BoneJ plugin of ImageJ.

### Diffusion kinetics

For diffusion kinetic study, fluorescein sodium salt was used as the model diffusing substance. Scaffolds were cut into discs (D × H = 8 × 5 mm) and submerged into 0.5 ml of 10 µg/ml aqueous solution of fluorescein sodium salt. The discs were taken out at 0.5 or 1.5 h and cut into half to study the cross-section. The fluorescent dye was excited using a 365 nm UV lamp and the images were taken using a Dino-lite digital microscope (USA).

### DPSCs isolation, flow cytometry and differentiation

Dental pulp stem cells were isolated from a third molar tooth extracted for clinical purposes with informed consent at Marquette University School of Dentistry. The procedure was approved by Marquette University graduate school. All research was performed in accordance with relevant guidelines/regulations and an exemption status was granted by the Institutional Review Board of Marquette University (Milwaukee, USA). The soft dental pulp tissue was removed from the root of the tooth after cutting the tooth using a dental drill bit. The pulp tissue was then transferred to a solution of 0.4%w/v Dispase II and 0.3%w/v Collagenase, Type I in PBS and kept in an incubator for 1–1.5 h. After addition of Minimum Essential Medium (MEM, Corning, Mediatech Inc., USA) supplemented with 10%v/v Fetal Bovine Serum (FBS, Sigma, USA) to the tube, the suspension was centrifuged, and the supernatant was replaced with complete media. The tissue debris and suspended cells were transferred to a small flask and cultured until the 4^th^ passage was reached.

For flow cytometric analysis, fresh cell suspensions (10^6^ cells/ml) were washed in blocking buffer (containing 3% BSA) and incubated with antibodies against FITC conjugated anti-CD90 (0.5 mg/ml) or FITC Mouse IgG1 Control, FITC anti-human CD44 (200 µl/ml) or FITC-conjugated mouse IgG2b control, Phycoerythrin (PE) anti-human CD34 (200 µl/ml) and PE anti-human CD45 (200 µl/ml) or PE-conjugated mouse IgG1 control for 45 min at 4 °C and resuspended in phosphate buffered saline (PBS). Attune acoustic focusing cytometer (Applied Biosystems, USA) was used to record forward and side scatters, identifying cell population and consequently recording the characteristics of the selected population.

For *In vitro* osteogenic differentiation, cells from passage 4 were seeded into 6-well plates (10,000 cells/well). Dulbecco’s modified Eagle’s medium (DMEM, Corning, Mediatech Inc., USA) supplemented with 100 nM dexamethasone, 0.05 M ascorbate-2-phosphate, 10 mM β-glycerophosphate, 1% antibiotic/antimycotic (Sigma, USA) and 10% FBS was added to the cells. After incubating for 3 weeks while replacing the medium twice a week, osteogenic differentiation was studied via Alizarin Red staining (EMD Millipore, USA). The cells were fixed using 10% neutral buffered formalin for 30 min, then stained with a 2% alizarin red-S solution at pH 4.2–4.4 at 37 °C for 10 min prior to imaging.

For adipogenic differentiation, cells of the 4^th^ passage were seeded into 6-well plates (20,000 cells/well) in adipogenic medium for 3 weeks. This media was supplemented with 10% FBS, 0.5 mM isobutyl-methyl-xanthine, 1 mM dexamethasone, 10 µg/ml insulin, 100 M indomethacin, and 1% antibiotic/antimycotic. The medium was replaced twice a week. The presence of intracellular lipid droplets was confirmed by Oil Red O staining (sigma, USA). For this purpose, the cells were fixed for 30 min using 10% neutral buffered formalin, and Oil Red O was applied for 5 min followed by observation under light microscope.

### Cell culture, cell attachment, histology and immunohistochemistry

Cells were cultured under standard aseptic conditions and used at passage 4 for cell seeding with a density of 10^6^ cells/cm2. The culture medium consisted of DMEM supplemented with 10% (v/v) FBS and 1% penicillin–streptomycin–amphotericin B. At day 7, scaffolds were taken out, washed with PBS and prepared for evaluating cell attachment and morphology. For SEM, samples were fixed in Karnovsky’s fixative (glutaraldehyde + paraformaldehyde) for 2 h, then fixed for 1 h using 1% Osmumium Tetroxide and then dehydrated using ethanol series (50, 70, 80, 95, 100% (v/v)) for 15–30 min each. After that, samples were dried chemically using Hexamethyldisilazane and left at room temperature for 24 h prior to gold sputter coating and imaging. PrestoBlue® (PB) cell vitality assay (Invitrogen, USA) was used to measure cell proliferation at different time intervals i.e. 3, 7 and 14 days according to the manufacturer’s instructions. At each time point, the media was replaced with 10% v/v Presto blue in phenol red free DMEM. The fluorescence intensities were measured at Excitation/Emission wavelengths of 540/590 nm using a microplate reader (Synergy HTX, BioTEK) after incubation for 1 h. the measurements were conducted in triplicate and the results were reported as mean ± SD. For histology, the scaffolds were stained using Hematoxylin and Eosin (H&E staining) through standard protocol. For this purpose, scaffolds were fixed in formalin, embedded in paraffin, sliced into 5–10 μm thick sections, and mounted on glass slides. The specimens were deparaffinized using xylenes and exposed to ethanol series. They were then stained through hematoxylin and eosin and dehydrated before cover-slipping. For immunofluorescence staining, F-actin Staining Kit-Cytopainter (abcam) was used after fixation in formalin and permeabilization of the cells. Green fluorescent phalloidin conjugate and 4′,6-diamidino-2-phenylindole dihydrochloride (DAPI) were used to stain the scaffolds and cell nuclei, respectively. Cell distribution was then, evaluated under a fluorescence microscope (Evos Flueroscent, life tecnologies).

## Electronic supplementary material

Supplementary Information

## References

[1] Lu T, Li Y, Chen T (2013). Techniques for fabrication and construction of three-dimensional scaffolds for tissue engineering. International Journal of Nanomedicine.

[2] Hollister SJ (2005). Porous scaffold design for tissue engineering. Nat Mater.

[3] Fierz FC (2008). The morphology of anisotropic 3D-printed hydroxyapatite scaffolds. Biomaterials.

[4] Rasoulianboroujeni M (2017). From solvent-free microspheres to bioactive gradient scaffolds. Nanomedicine: Nanotechnology, Biology and Medicine.

[5] Cao L, Wang J, Hou J, Xing W, Liu C (2014). Vascularization and bone regeneration in a critical sized defect using 2-N, 6-O-sulfated chitosan nanoparticles incorporating BMP-2. Biomaterials.

[6] Mohanty S (2016). Fabrication of scalable tissue engineering scaffolds with dual-pore microarchitecture by combining 3D printing and particle leaching. Materials Science and Engineering: C.

[7] Morgan KY (2016). Multi-Material Tissue Engineering Scaffold with Hierarchical Pore Architecture. Advanced functional materials.

[8] Harris, J. N., Ling, J. & Cheng, X. Google Patents, (2017).

[9] Gao, Y. M. *et al*. Biocompatible 3D Liquid Crystal Elastomer Cell Scaffolds and Foams with Primary and Secondary Porous Architecture. A*CS Publications* (2016). 10.1021/acsmacrolett.5b00729 35668595

[10] Zhang C, Wen X, Vyavahare NR, Boland T (2008). Synthesis and characterization of biodegradable elastomeric polyurethane scaffolds fabricated by the inkjet technique. Biomaterials.

[11] Park, K. *et al*. InM*acromolecular Symposia*. 145–150 (Wiley Online Library).

[12] Morais AR (2016). Freeze-drying of emulsified systems: A review. International journal of pharmaceutics.

[13] Liu H, Nakagawa K, Chaudhary D, Asakuma Y, Tadé MO (2011). Freeze-dried macroporous foam prepared from chitosan/xanthan gum/montmorillonite nanocomposites. Chemical Engineering Research and Design.

[14] S, H. M., A, P. R. & Marvin, S. Google Patents (1969).

[15] Angulo DEL, Sobral PJDA (2016). The Effect of Processing Parameters and Solid Concentration on the Microstructure and Pore Architecture of Gelatin-Chitosan Scaffolds Produced by Freeze-Drying. Materials Research.

[16] Konstantinidis AK, Kuu W, Otten L, Nail SL, Sever RR (2011). Controlled nucleation in freeze-drying: effects on pore size in the dried product layer, mass transfer resistance, and primary drying rate. Journal of pharmaceutical sciences.

[17] Schoof H, Apel J, Heschel I, Rau G (2001). Control of pore structure and size in freeze-dried collagen sponges. Journal of Biomedical Materials Research.

[18] Ni X (2016). The control of ice crystal growth and effect on porous structure of konjac glucomannan-based aerogels. International journal of biological macromolecules.

[19] Prins, A., Bos, M. A., Boerboom, F. J. G. & van Kalsbeek, H. K. A. I. In *Studies in Interface Science* Vol. Volume 7 (eds Möbius Dietmar & Miller Reinhard) 221–265 Elsevier, (1998).

[20] Yohko FY (2012). Kinetics of protein unfolding at interfaces. Journal of Physics: Condensed Matter.

[21] Dickinson E (1999). Caseins in emulsions: interfacial properties and interactions. International Dairy Journal.

[22] Surh J, Decker EA, McClements DJ (2006). Properties and stability of oil-in-water emulsions stabilized by fish gelatin. Food Hydrocolloids.

[23] Toledano O, Magdassi S (1998). Emulsification and Foaming Properties of Hydrophobically Modified Gelatin. Journal of Colloid and Interface Science.

[24] Zarai Z, Balti R, Sila A, Ben Ali Y, Gargouri Y (2016). Helix aspersa gelatin as an emulsifier and emulsion stabilizer: functional properties and effects on pancreatic lipolysis. Food & Function.

[25] Lobo L (2002). Coalescence during Emulsification. Journal of Colloid and Interface Science.

[26] Halling PJ (1981). Protein-stabilized foams and emulsions. Critical reviews in food science and nutrition.

[27] Saint-Jalmes A, Peugeot ML, Ferraz H, Langevin D (2005). Differences between protein and surfactant foams: Microscopic properties, stability and coarsening. Colloids and Surfaces A: Physicochemical and Engineering Aspects.

[28] Brewer DR, Franco JM, Garcia-Zapateiro LA (2016). Rheological properties of oil-in-water emulsions prepared with oil and protein isolates from sesame (Sesamum Indicum). Food Science and Technology (Campinas).

[29] Saint-Jalmes A (2006). Physical chemistry in foam drainage and coarsening. Soft Matter.

[30] Im O, Li J, Wang M, Zhang LG, Keidar M (2012). Biomimetic three-dimensional nanocrystalline hydroxyapatite and magnetically synthesized single-walled carbon nanotube chitosan nanocomposite for bone regeneration. Int J Nanomedicine.

[31] Shahini A (2014). 3D conductive nanocomposite scaffold for bone tissue engineering. International Journal of Nanomedicine.

[32] Li X, Qin J, Ma J (2015). Silk fibroin/poly (vinyl alcohol) blend scaffolds for controlled delivery of curcumin. Regenerative Biomaterials.

[33] Pav?n, J. *et al*. Advanced titanium scaffolds obtained by directional freeze-drying: on the influence of processing conditions. *Frontiers in Bioengineering and Biotechnology*, 10.3389/conf.FBIOE.2016.01.03006.

[34] Ali M (2014). A comparison study of different physical treatments on cartilage matrix derived porous scaffolds for tissue engineering applications. Science and Technology of Advanced Materials.

[35] Izquierdo-López D, Goulet J, Ratti C (2017). Foam-Mat Freeze-Drying of Bifidobacterium longum RO175: Viability and Refrigerated Storage Stability. Journal of Food Science.

[36] Morgan KY (2016). Multi‐Material Tissue Engineering Scaffold with Hierarchical Pore Architecture. Advanced functional materials.

[37] Blunt, M. J. *Multiphase flow in permeable media: a pore-scale perspective*. Cambridge University Press, (2017).

[38] Odgaard A, Gundersen H (1993). Quantification of connectivity in cancellous bone, with special emphasis on 3-D reconstructions. Bone.

[39] Tellis BC (2009). Trabecular scaffolds created using micro CT guided fused deposition modeling. Materials science & engineering. C, Materials for biological applications.

[40] Grayson WL (2008). Effects of Initial Seeding Density and Fluid Perfusion Rate on Formation of Tissue-Engineered Bone. Tissue engineering. Part A.

[41] Dong Y, Wu D, Chen X, Lin Y (2010). Adsorption of bisphenol A from water by surfactant-modified zeolite. Journal of colloid and interface science.

[42] Kuo C-Y (2009). Comparison with as-grown and microwave modified carbon nanotubes to removal aqueous bisphenol A. Desalination.

[43] Murphy CM, Haugh MG, O'Brien FJ (2010). The effect of mean pore size on cell attachment, proliferation and migration in collagen–glycosaminoglycan scaffolds for bone tissue engineering. Biomaterials.

[44] Whang K (1999). Engineering bone regeneration with bioabsorbable scaffolds with novel microarchitecture. Tissue engineering.

[45] Yang S, Leong K-F, Du Z, Chua C-K (2001). The design of scaffolds for use in tissue engineering. Part I. Traditional factors. Tissue engineering.

[46] Zeltinger J, Sherwood JK, Graham DA, Müeller R, Griffith LG (2001). Effect of pore size and void fraction on cellular adhesion, proliferation, and matrix deposition. Tissue Engineering.

[47] Artel A, Mehdizadeh H, Chiu Y-C, Brey EM, Cinar A (2011). An agent-based model for the investigation of neovascularization within porous scaffolds. Tissue Engineering Part A.

[48] Kuboki Y, Jin Q, Takita H (2001). Geometry of carriers controlling phenotypic expression in BMP-induced osteogenesis and chondrogenesis. JBJS.

[49] Van Tienen TG (2002). Tissue ingrowth and degradation of two biodegradable porous polymers with different porosities and pore sizes. Biomaterials.

