# Supplementary material for: Dual Porosity Protein-based Scaffolds with Enhanced Cell Infiltration and Proliferation
Source: Sci Rep. 2018 Oct 5;8:14889. doi: 10.1038/s41598-018-33245-w (PMC6173780; doi:10.1038/s41598-018-33245-w)
Supplement: Supplementary file 1 — Supplementary Information [file 41598_2018_33245_MOESM1_ESM.docx]

**Dual porosity protein-based scaffolds with enhanced cell infiltration and proliferation**

Morteza Rasoulianboroujeni^1,2,*^, Nasim Kiaie^3^, Fahimeh Sadat Tabatabaei^1, 4^, Amir Yadegari^1^, Farahnaz Fahimipour^1^, Kimia Khoshroo^1^, Lobat Tayebi^1,*^

^1^ Marquette University School of Dentistry, Milwaukee, WI, USA

^2^ Division of Pharmaceutical Sciences, School of Pharmacy, University of Wisconsin-Madison, Madison, WI, USA

^3^ Department of Tissue Engineering, Amirkabir University of Technology, Tehran, Iran

^4^ Department of dental biomaterials, School of dentistry, Shahid Beheshti University of Medical Sciences, Tehran, Iran

* Corresponding author:

Morteza Rasoulianboroujeni

Marquette University School of Dentistry, Milwaukee, WI, USA

Email: [Morteza.rasoulianboroujeni@marquette.edu](mailto:Morteza.rasoulianboroujeni@marquette.edu)

Lobat Tayebi

Marquette University School of Dentistry, Milwaukee, WI, USA

Email: [lobat.tayebi@marquette.edu](mailto:lobat.tayebi@marquette.edu)

**1. Experimental procedure**

**1.1. Rheological characterization**

Different concentrations of gelatin aqueous solution (5%, 10%, 15% (w/v)) were prepared by dissolving gelatin in DI water at 50°C. Each solution was agitated at 500 or 1500 rpm using a mechanical mixer (IKA, USA) for 15 min while maintaining the temperature at 40°C. The rheological properties of the obtained gelatin foams or the corresponding solutions were investigated using a Kinexus shear rheometer (Malvern, UK) using a stainless-steel cone-plate geometry (D = 40 mm). A Peltier temperature controller was used to adjust the temperature throughout the experiments. An adequate amount of gelatin solution or foam was loaded onto the pre-cleaned Peltier plate of the rheometer and the upper plate was gently lowered until it touched the sample. The oscillatory shear measurements and viscometery were conducted at various temperatures ranging from 22 to 37 °C. In the oscillation mode, for each temperature, different frequencies ranging from 1 to 15 Hz were scanned at a constant shear strain of 2% corresponding to the angular displacement of 4 × 10^−4^ rad to ensure remaining in the linear viscoelastic regions where the loss and storage modulus are independent of the strain amplitude. The viscometry consisted of two consecutive shear cycles at each specific temperature. The shear rate varied logarithmically in ramp mode from 0 to 50 s^−1^ and then back to 0 s^−1^. The kinetics of aging was studied by oscillating the samples at 5 Hz and 0.5% shear strain. The storage, loss and complex moduli for each sample were monitored over a 30 min period.

**1.2. FTIR Spectroscopy**

The FTIR spectra of uncross-linked and cross-linked samples were collected using ATR-FTIR instrument (Nicolet iS5 Thermo Scientific^TM^ USA, OMNICTM). To investigate the functional vibration groups of pristine and cross-linked porcine gelatin-based scaffolds, each spectrum was collected over the wavelength range of 500-4000 cm^-1^ with the resolution of 4 cm^-1^ and the accumulation of 32 scans.

**2. Results and Discussion**

**2.1. Rheological characterization**

According to the method proposed by Winter and Chambon [1, 2], the gelation temperature can be defined as the temperature at which dissipation factor or Tan delta (loss tangent) is frequency-independent [3]. It is obvious that for the thermos-reversible gels like gelatin, temperature is the most important factor which should be controlled. The gelation temperatures of gelatin solutions and foams at three concentrations were determined through plotting the dissipation factor as a function of temperature at different frequencies (Fig. S1). According to Fig. S1, the gelation temperature increased by increasing the concentration of gelatin. The gelation temperature of 5, 10, and 15% w/v gelatin solution were estimated to be 24, 27, and 29 °C, respectively. gel point is directly proportional to the interactions between chains. The gelation occurs through local assembly of protein coils into helices dominated by hydrogen bonding. During the gelation process, the coil-to-helix transition occurs through which the gelatin chains are prone to recover the collagen triple-helix structure. These transitions are responsible for formation of physical interactions between the gelatin chains and ultimately gelation [3]. Hence, it is obvious that by increasing the concentration of gelatin solution the percolation of physical junctions and entanglement of gelatin chains occur at higher temperature. As seen in Fig S1, the gelation temperature of gelatin foam samples at 500 rpm increased by increasing the concentration of gelatin, indicating a similar behavior as gelatin solutions. The gelation temperature of 5, 10, and 15% w/v gelatin foamed at 500 rpm were estimated to be 27, 28, and 30 °C, respectively. Interestingly, at a cetrain concentration, foaming at 500 rpm resulted in elevated gelation temperatures. Foaming at 1500 rpm further increased the gelation temperature at each concentration examined. All in all, the augmentation of agitation speed led to increase of the gelation temperature at all concentrations. However, at higher concentrations i.e. 10 and 15% w/v, such effect was less significant than 5% w/v gelatin foam.

The viscosity of the gelatin solutions and foam samples as a function of shear rate was also investigated at different temperatures. For this purpose, the shear viscosity was plotted against shear rate at various temperatures ranging from 22 to 36 °C. Figure S2 shows the viscosity plots for various concentrations of


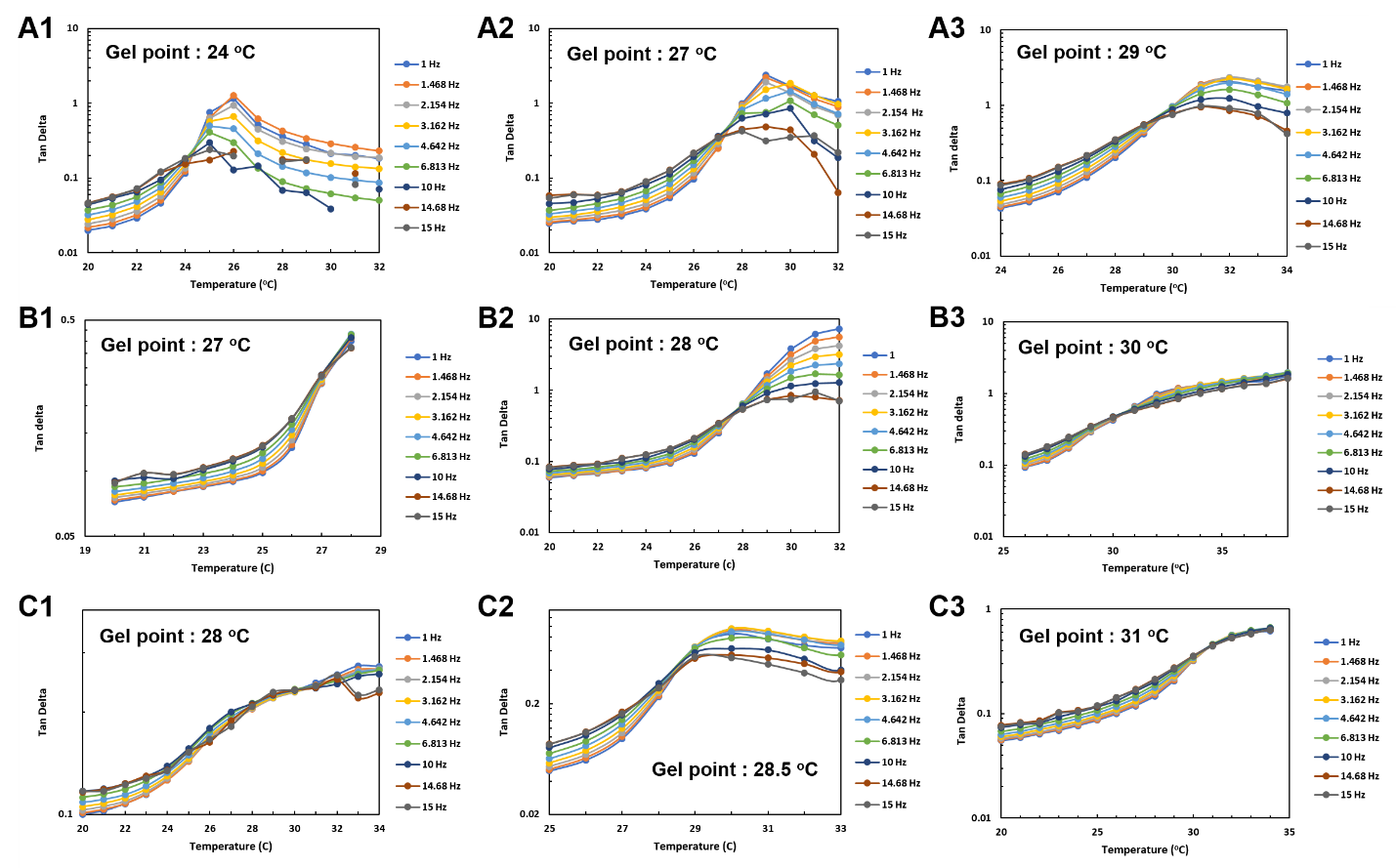


Figure S1. The loss tangent of (A) gelatin solutions, (B) gelatin foamed at 500 rpm and (C) gelatin foamed at 1500 rpm vs temperature in various frequencies. Different concentrations of gelatin i.e. (A1-C1) 5, (A2-C2) 10, and (A3-C3) 15% w/v were examined.

gelatin solutions and foams. As seen, at all the concentrations viscosity decreased with increasing temperature from 22 to 34 °C. According to the graphs at different concentrations and low temperatures, the gelatin solutions and foams represent a shear-thinning behavior. The non-Newtonian behavior is more evident at low shear rates. However, at higher shear rate the samples reveal almost Newtonian fluid behavior [4]. At higher temperature, the samples represented Newtonian behavior both at low and high shear rates. The viscosity of the hydrogels highly depends on temperature specially near the gel point. The higher viscosity of the solutions is due to conducting the experiments at temperatures below gelation point. For instance, in Fig S2A1, the value of viscosity at 22 °C which is below the gelation temperature (i.e. 24 °C) is significantly higher than those values at higher temperatures. Figures S2A2 and A3 show the plots of shear rate versus viscosity for the gelatin solutions at concentrations of 10 and 15 % w/v, respectively. As seen, in Fig S2A2, the values of viscosity are higher at 22, 24, and 26 °C which are below the gelation temperature (27 °C). Figure S2B and C show the viscosity of gelatin (5, 10, and 15 % w/v) foamed at 500 and 1500 rpm, respectively. As presented, the shear viscosity of the gelatin foams follow a similar trend as observed for gelatin solutions. Foaming seems not to have a significant impact on the overall behavior of gelatin in terms of viscosity.

Time dependency of the storage and loss modulus was measured to investigate the aging process of the samples. The kinetics of aging is expected to influence the values of loss and storage moduli over time. Time dependency of loss, storage and complex moduli was monitored at 37 °C over a fixed time period and


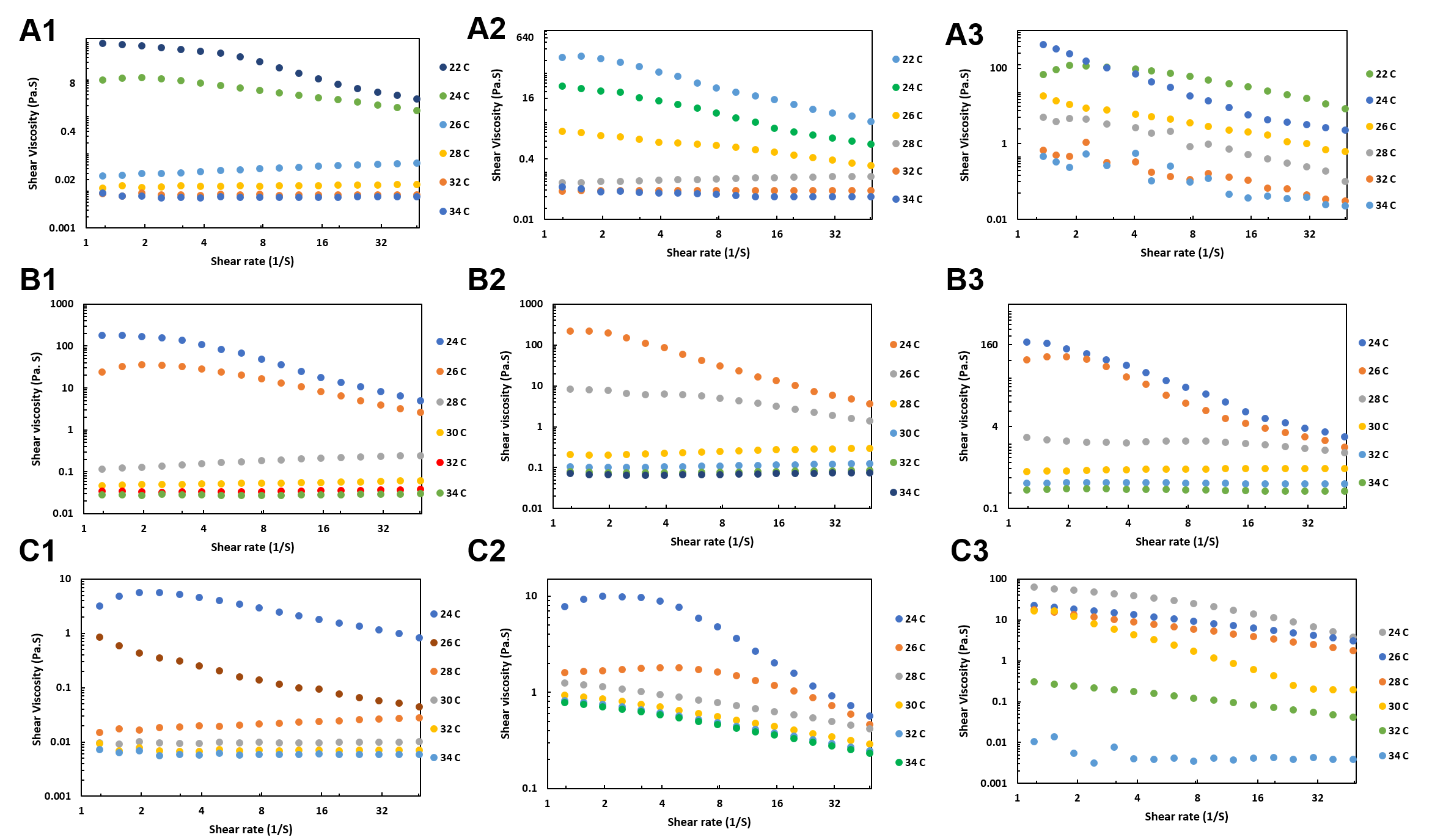


Figure S2. The viscosity of (A) gelatin solutions, (B) gelatin foamed at 500 rpm and (C) gelatin foamed at 1500 as a function of shear rate in various temperatures. Different concentrations of gelatin i.e. (A1-C1) 5, (A2-C2) 10, and (A3-C3) 15% w/v were examined.

constant frequency of 5 Hz. Figure S3 shows the time dependency of storage, loss, and complex moduli of different concentrations of gelatin solution. As seen, the moduli of 5% w/v gelatin solution remains almost unchanged (Fig S3A). Interestingly, the moduli increased with increasing the concentration of gelatin. The moduli increased at a higher slope for 15% w/v compared to 10% w/v. Overall, the changes in the moduli of solutions over time were negligible. The time dependency of moduli of different concentrations of gelatin foamed at 500 and 1500 rpm are depicted in Fig. S4 and S5, respectively. As illustrated, foaming resulted in a significant rise in the initial value of moduli. According to Fig. S4, foaming at 500 rpm left a slight change in the moduli of 5 and 10% w/v samples but enlarged the 15% w/v sample moduli significantly. Interestingly, 5 and 10% samples displayed little transient or no time dependency, but 15% sample exhibited an absolute time-dependent behavior. Such as observation can be attributed to the insufficiancy of agitation at 500 rpm to produce desired foams aout of 5 and 10% w/v gelatin solutions. Such as assumption is in agreement with the unfavorable structures observed in SEM images (Figure 1) when certain concentrations and speeds were used to produce scaffolds. in contrast, foaming at 1500 rpm resulted in a time-dependent behavior where loss, storage, and complex moduli of all the samples exponentially decreased over time. Someone may remark the unfavaroble structure of scaffolds prepared using 5% w/v gelatin foamed at 1500 rpm. It should be noted that the foam produced using the mentioned parameters is very unstable, as seen in Fig. S5, and aging may result in complete separation of liquid and foam.


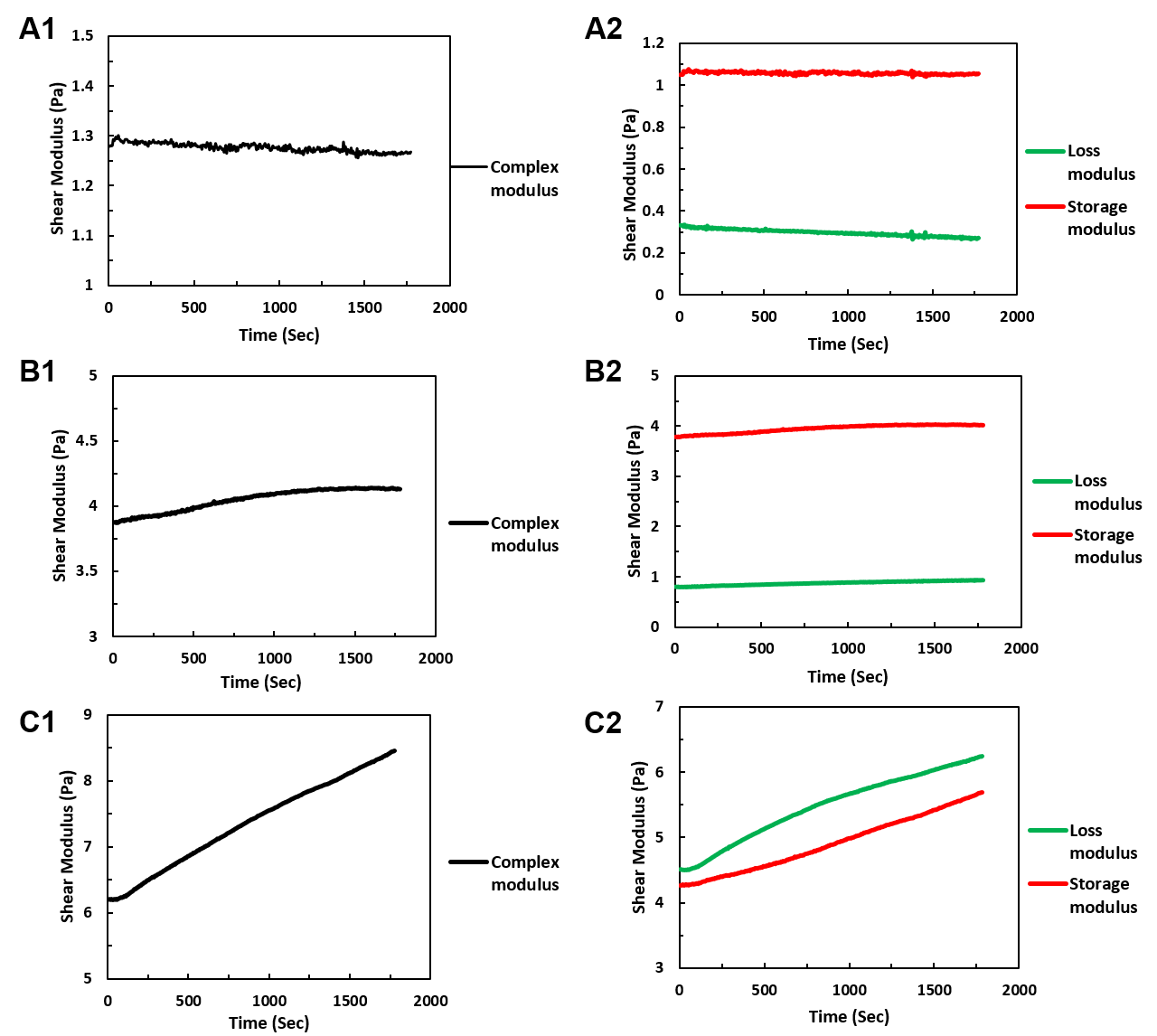


Figure S3. The aging process of (A) 5, (B) 10, and (C) 15% w/v gelatin solutions investigated through monitoring the variation of complex (A1-C1), storage and loss (A2-C2) moduli over a 30 min period.


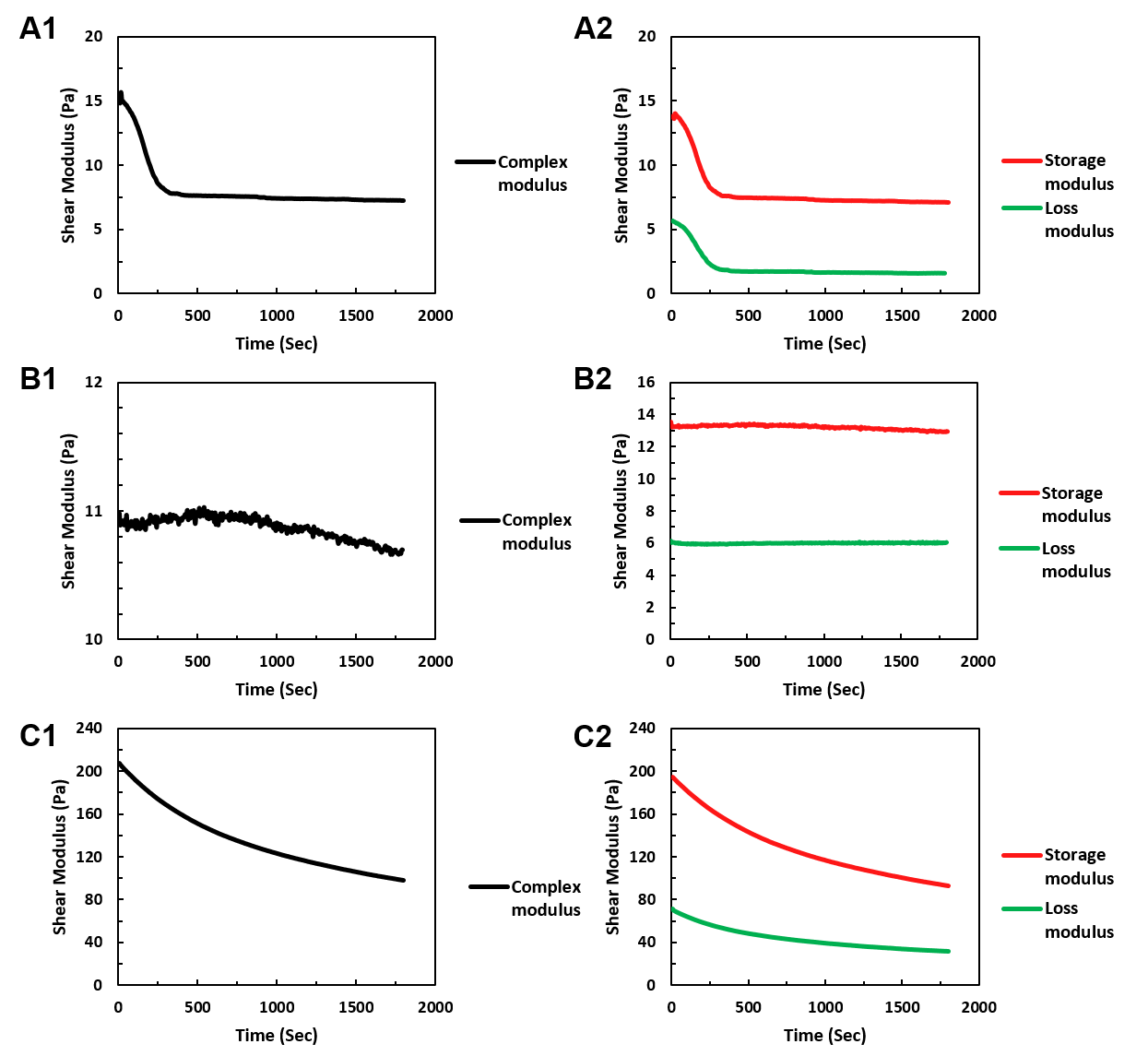


Figure S4. The aging process of (A) 5, (B) 10, and (C) 15% w/v gelatin foamed at 500 rpm investigated through monitoring the variation of complex (A1-C1), storage and loss (A2-C2) moduli over a 30 min period.


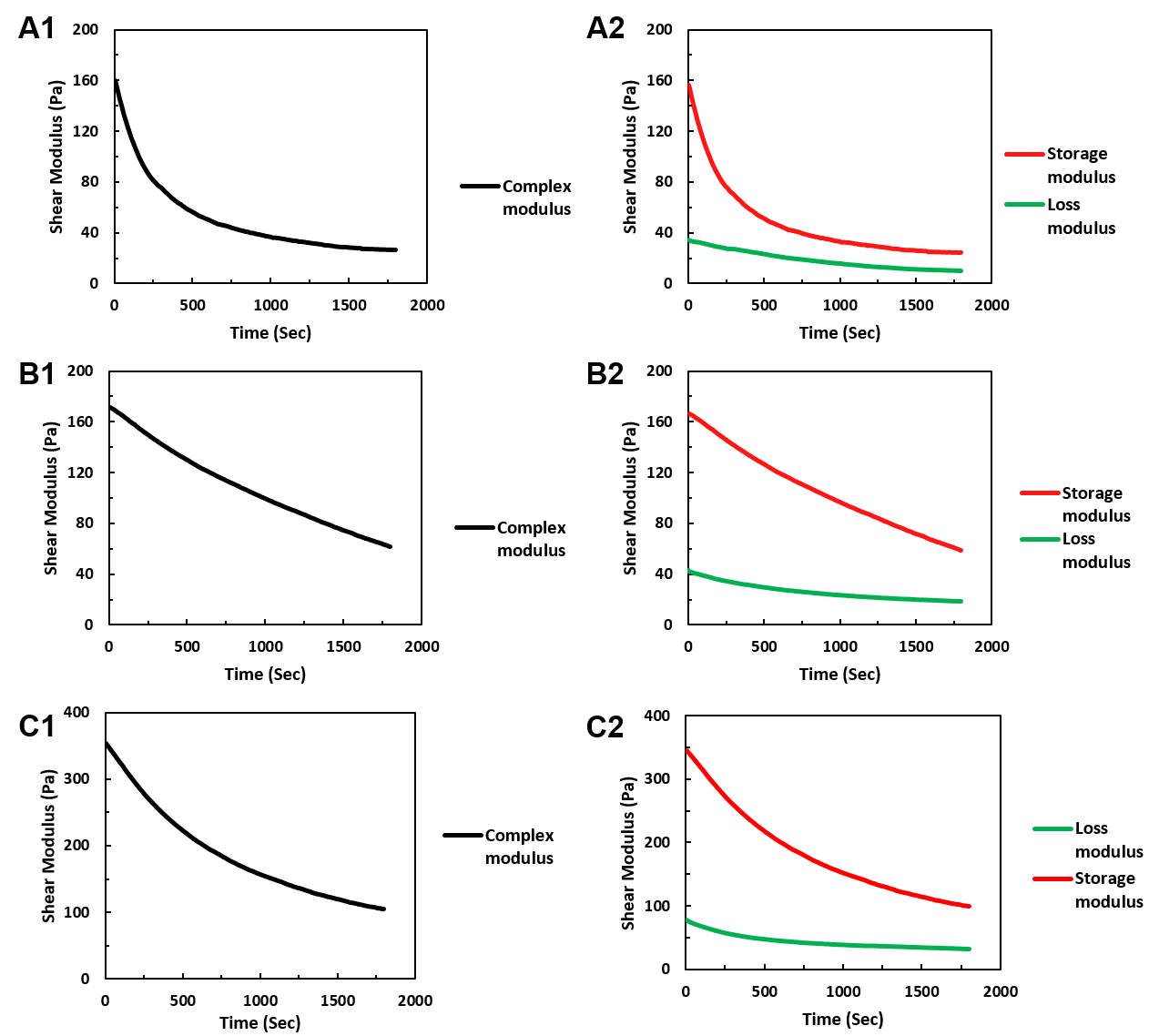


Figure S5. The aging process of (A) 5, (B) 10, and (C) 15% w/v gelatin foamed at 1500 rpm investigated through monitoring the variation of complex (A1-C1), storage and loss (A2-C2) moduli over a 30 min period.

**2.2. FTIR spectroscopy**

According to Fig S6, the FTIR spectra of both pristine porcine gelatin scaffolds and gelatin scaffolds cross-linked via EDC-NHS represent similar spectral characteristics suggesting the creation of no new bond. However, the intensity of some peaks has been changed suggesting successful cross linking. The distinctive peaks at 1000-1100 cm^-1^ can be attributed to the characteristic absorption of C-O and C-O-C bonds. The sharp peak at 1030 cm^-1^, assigned to C-O stretching, can be observed in pristine gelatin scaffold spectrum while it has disappeared after cross-linking implying the reaction of carboxylic groups [5]. Furthermore, the weak peak at 1081 cm^-1^ can be attributed to the presence of carbohydrate residues in collagen [6]. The typical peaks at 1236 cm^-1^ can be ascribed to C-N stretching vibrations and N-H bending vibrations which are generally corresponding to Amide III [7]. It should be noted that the weak bending vibrations of C-C and C=O could occur at this wavelength. It has been also proposed that protein secondary structure can be realized according to Amide III absorption band [8]. The distinctive peaks at 1455 and 1538 cm^-1^ are the characteristics of N-H bending and C-N stretching vibrations corresponding to Amide II [8]. The strong peaks at 1645 cm^-1^ is attributed to stretching vibrations C=O bonds in Amide II [8]. Interestingly, the intensity of all the amide II related peaks increased after cross-linking that is, in fact, due to formation of new amide II groups through reaction of carboxylic and amine groups of gelatin chains. The peaks at 2940, 3059, and 3311 cm^-1^ are related to symmetric aliphatic C-H groups, asymmetric C-H, and O-H bonds, respectively.


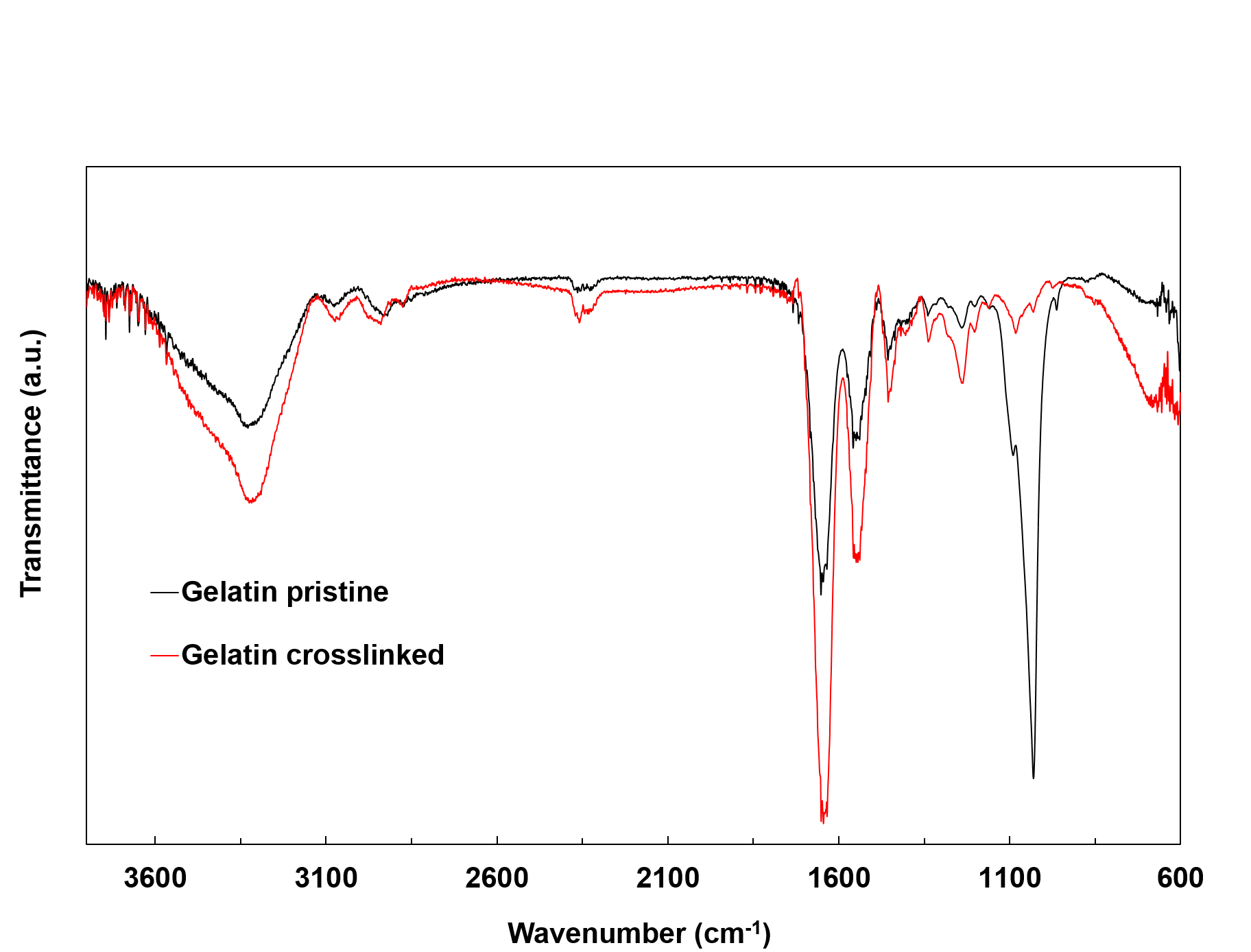


Figure S6. FT-IR spectra of pristine and cross-linked porcine gelatin-based scaffold

**2.3. Scaffold architecture**

Using the suggested method, large scaffolds with negligible shrinkage can be fabricated (Figure S7). Fabricating a large scaffold alone is not enough for successful regeneration as such template is required to ensure suitable nutrients and oxygen supply for the core part. Doing so, cells can migrate to deep regions of the scaffold. Due to insufficient oxygen and nutrient transport within the deep compartments of conventional scaffolds, the maximum cell penetration depth has been 150–240 µm [9].


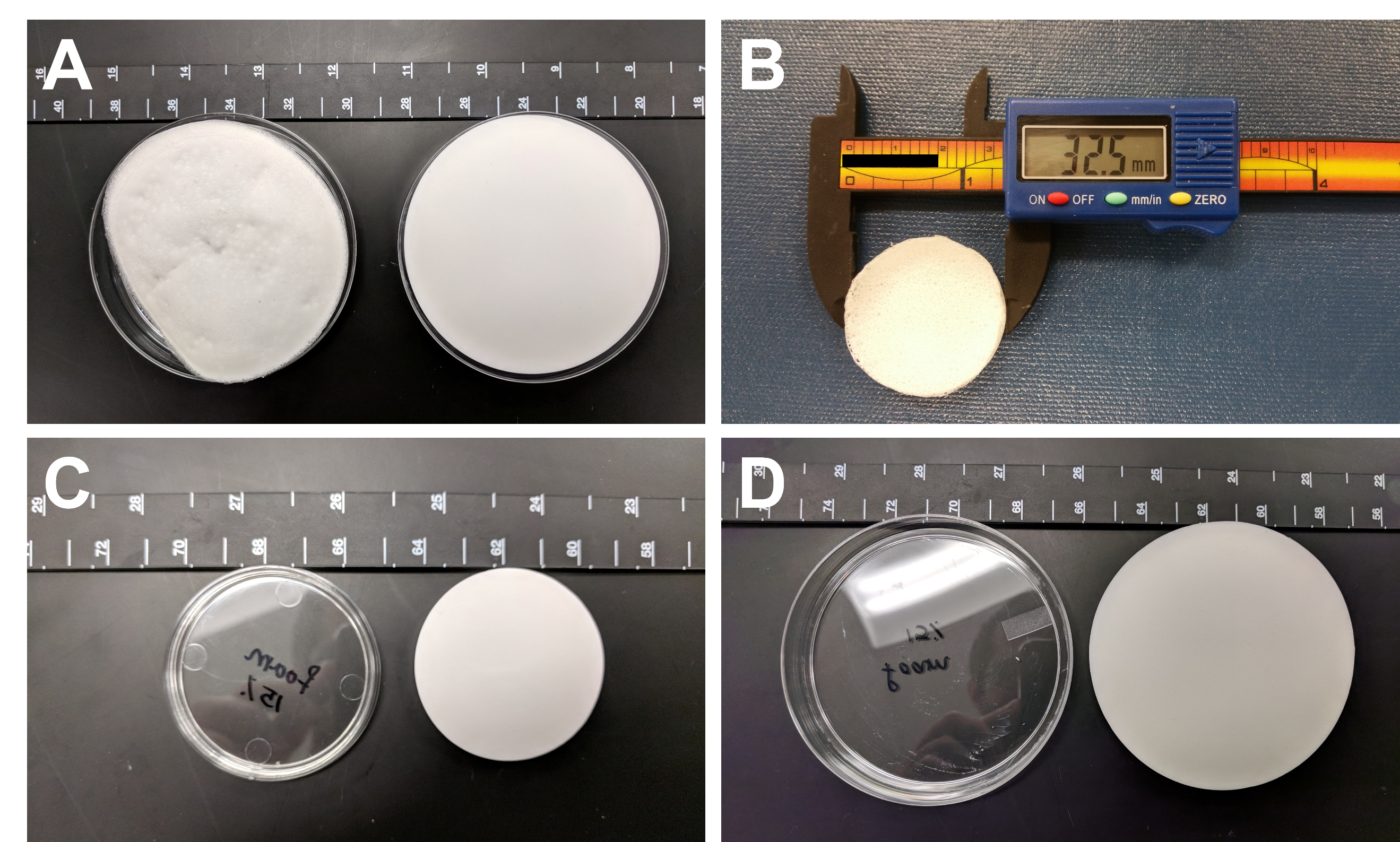


Figure S7. The effect of fabrication procedure on shrinkage and deformation. (A) conventional scaffold (left) vs. dual network scaffold (right). The negligible shrinkage of dual network scaffold when a (B) small i.e. D=33 mm, (C) medium i.e. D=50 mm or (C) big i.e. D=85 mm mold is used.

The reported design/structure in this study is expected to improve the transport of nutrients and waste within the scaffold through its unique feature, existence of small pores on the surface of bigger pores. The dual interconnected network of pores can facilitate concurrent nutrient supply and tissue ingrowth. The suggested scaffold exhibits uniform distribution of primary pores as the formation of primary network is controlled by the bubbling process rather than non-uniform development of the ice crystals [10]. Agitation speed, time and temperature along with gelatin concentration can influence the primary structure. Secondary structure, on the other hand, is formed through rapid freezing of the water in the walls of the bubbles and sublimation of the resulting ice crystals. Facilitated heat transfer owing to high surface area of the bubbles, results in formation of small and uniform ice crystals thru the freezing step which in turn brings a fine secondary structure. The comparison between the conventional and dual network scaffolds has been schematically represented in Figure S8.


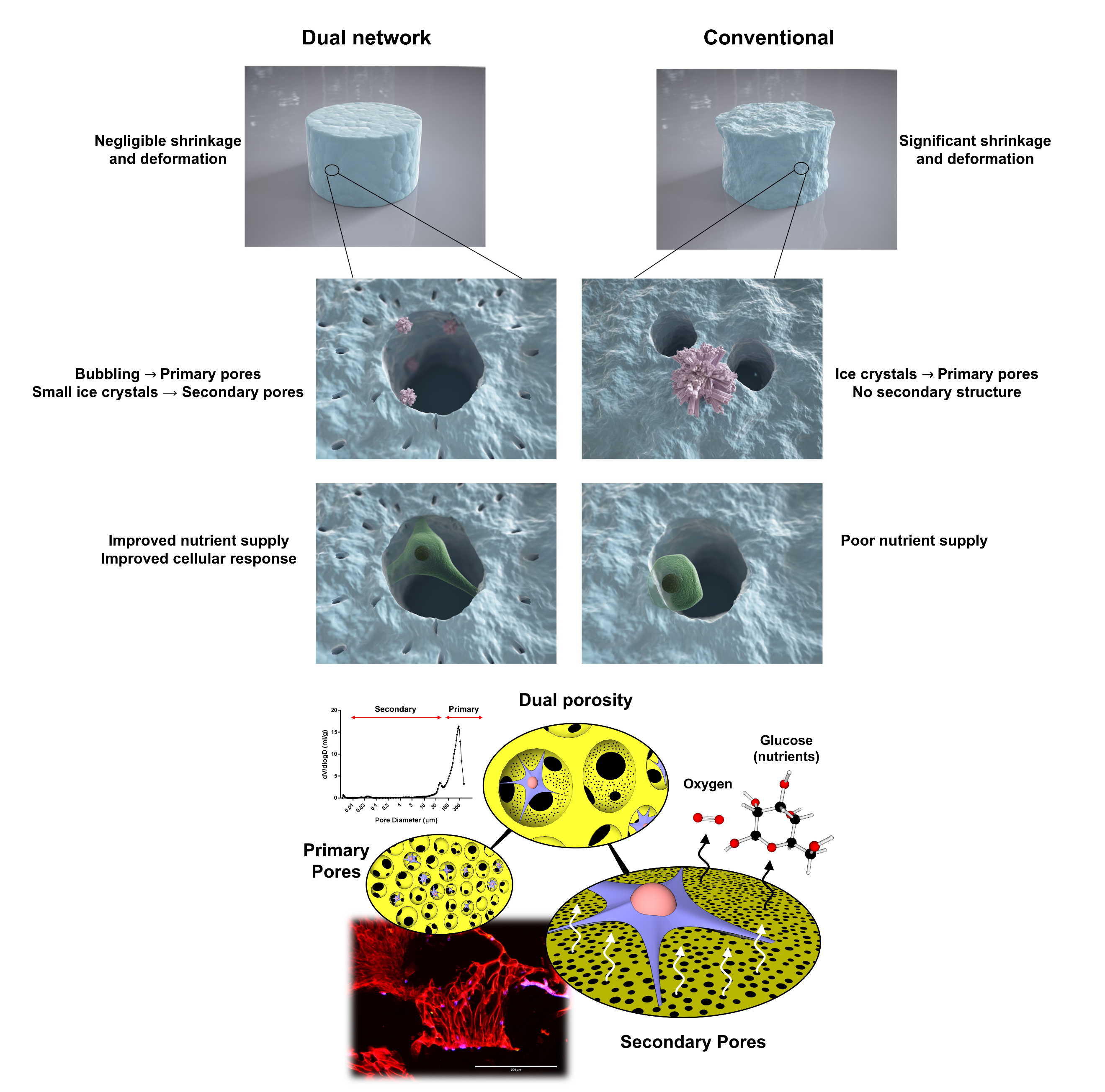


Figure S8. Schematic comparison between dual network and conventional scaffolds.

The majority of primary pores fall within the range of 100-300 µm for our scaffold which is good for osteogenesis (Figure S9). An interesting finding is that scaffold has macro, micro, and nanopores. This helps imitate cell microenvironment. Very small pores allow for transport of nutrition and oxygen, waste removal, and signaling; medium pores (∼5-15 µm) facilitate fibroblast cell infiltration, proliferation and capillary formation; pores of 30 to 40 are suitable for the regeneration of capillaries and big pores help incorporation of nerves and blood vessels. It has been suggested that cell spreading, initial cell proliferation, cell differentiation as well as the amount of ECM production all are affected by the pore size and porosity of scaffold [11-16].


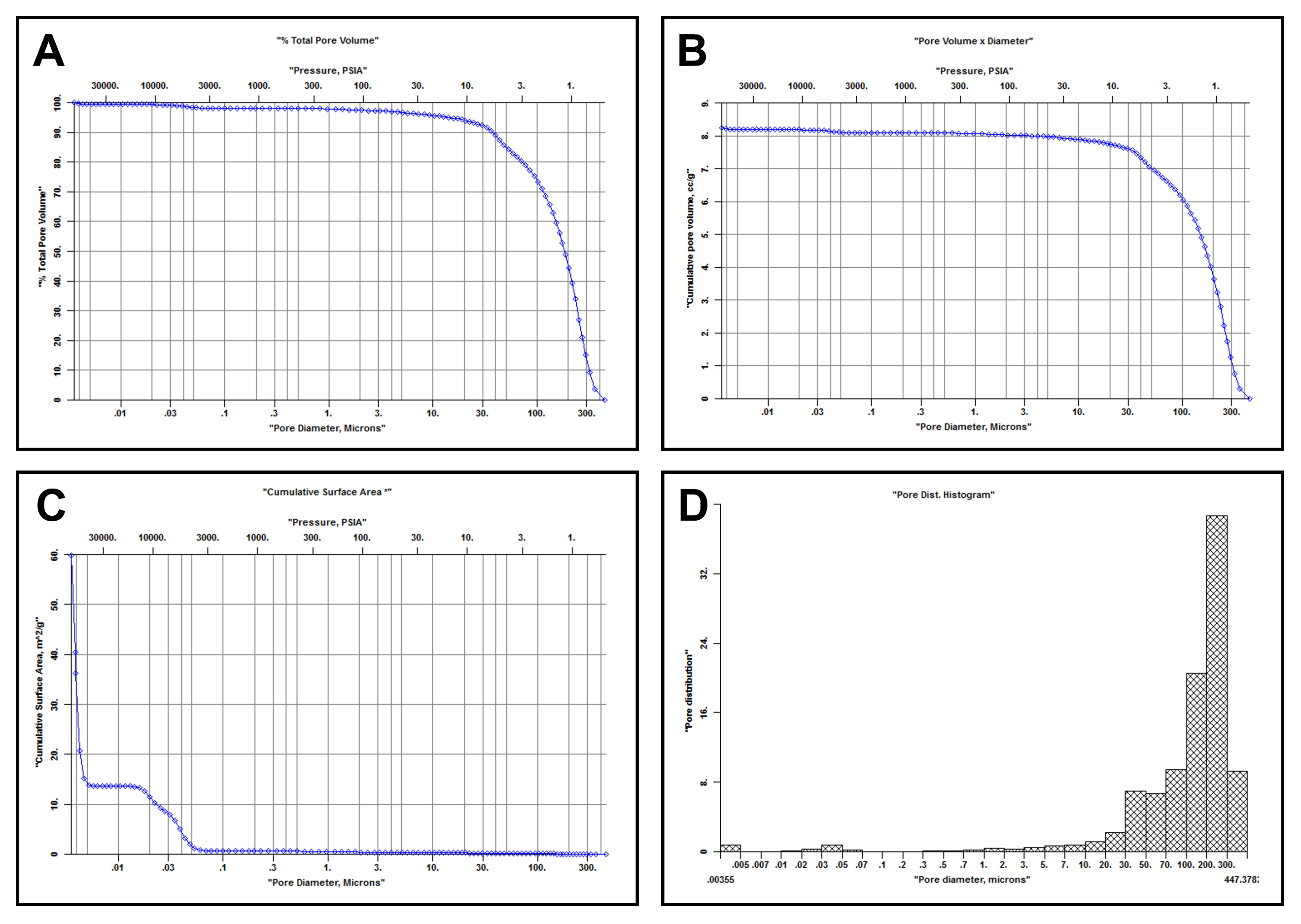


Figure S9. Mercury intrusion porosimetry results for the dual network scaffold: (A) cumulative percentage of total pore volume vs. pore diameter/pressure; (B) cumulative pore volume (ml/g) vs. pore diameter/pressure; (C) cumulative surface area (m^2^/g) vs. pore diameter/pressure; (D) pore size distribution.

**2.4. Isolation of Dental Pulp Stem Cells**

Flow cytometry illustrated that the isolated cells were positive for CD44, CD90 and CD 105, while being negative for hematopoietic markers of CD34 and CD45 (Figure S10). The obtained results suggest that the isolated cells were dental pulp stem cells.


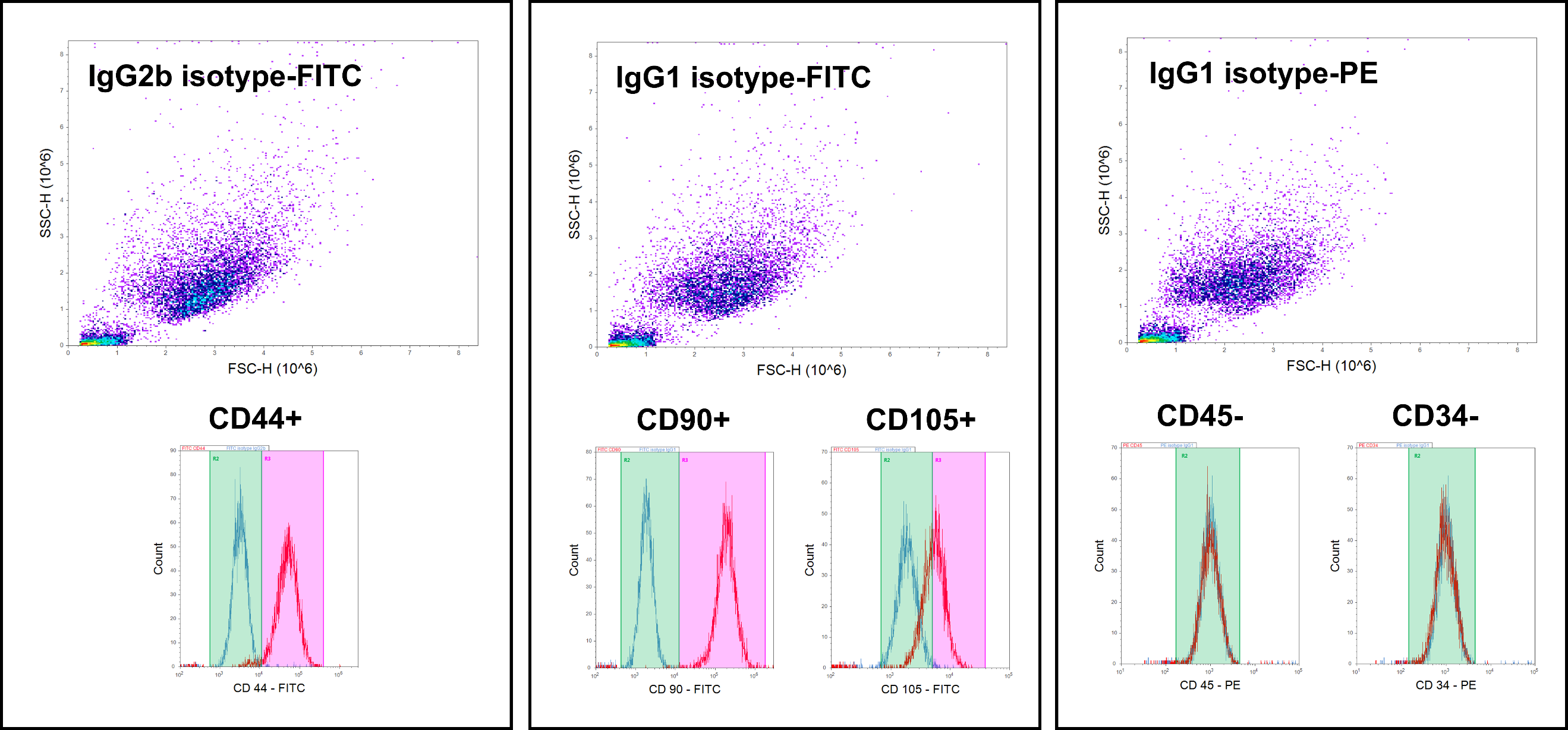


Figure S10. Flow cytometry analysis confirmed the isolation of dental pulp stem cells.

**3. References**

[1] Winter H. Can the gel point of a cross‐linking polymer be detected by the G′–G ″crossover? Polymer Engineering & Science 1987;27:1698-702.

[2] Winter H, Chambon F. Rheol. 1986, 30, 367. CrossRef| CAS| Web of Science® Times Cited;1138.

[3] Ross-Murphy SB. Structure and rheology of gelatin gels: recent progress. Polymer 1992;33:2622-7.

[4] Tanaka F. Polymer Physics: Applications to Molecular Association and Thermoreversible Gelation: Cambridge University Press; 2011.

[5] Belbachir K, Noreen R, Gouspillou G, Petibois C. Collagen types analysis and differentiation by FTIR spectroscopy. Analytical and bioanalytical chemistry 2009;395:829-37.

[6] Cebi N, Durak MZ, Toker OS, Sagdic O, Arici M. An evaluation of Fourier transforms infrared spectroscopy method for the classification and discrimination of bovine, porcine and fish gelatins. Food chemistry 2016;190:1109-15.

[7] Bandekar J. Amide modes and protein conformation. Biochimica et Biophysica Acta (BBA)-Protein Structure and Molecular Enzymology 1992;1120:123-43.

[8] Jackson M, Mantsch HH. The use and misuse of FTIR spectroscopy in the determination of protein structure. Critical reviews in biochemistry and molecular biology 1995;30:95-120.

[9] Botchwey EA, Dupree MA, Pollack SR, Levine EM, Laurencin CT. Tissue engineered bone: measurement of nutrient transport in three-dimensional matrices. Journal of biomedical materials research Part A 2003;67:357-67.

[10] Liu H, Nakagawa K, Chaudhary D, Asakuma Y, Tadé MO. Freeze-dried macroporous foam prepared from chitosan/xanthan gum/montmorillonite nanocomposites. Chemical Engineering Research and Design 2011;89:2356-64.

[11] Brauker JH, Carr-Brendel VE, Martinson LA, Crudele J, Johnston WD, Johnson RC. Neovascularization of synthetic membranes directed by membrane microarchitecture. J Biomed Mater Res 1995;29:1517-24.

[12] Klawitter JJ, Hulbert SF. Application of porous ceramics for the attachment of load bearing internal orthopedic applications. Journal of Biomedical Materials Research 1971;5:161-229.

[13] Whang K, Healy KE, Elenz DR, Nam EK, Tsai DC, Thomas CH, et al. Engineering bone regeneration with bioabsorbable scaffolds with novel microarchitecture. Tissue engineering 1999;5:35-51.

[14] Loh QL, Choong C. Three-Dimensional Scaffolds for Tissue Engineering Applications: Role of Porosity and Pore Size. Tissue Engineering Part B, Reviews 2013;19:485-502.

[15] Sosnowski S, Wozniak P, Lewandowska-Szumiel M. Polyester scaffolds with bimodal pore size distribution for tissue engineering. Macromolecular bioscience 2006;6:425-34.

[16] Loh QL, Choong C. Three-dimensional scaffolds for tissue engineering applications: role of porosity and pore size. Tissue Eng Part B Rev 2013;19:485-502.
